# A Comprehensive Bioinformatics Analysis of Notch Pathways in Bladder Cancer

**DOI:** 10.3390/cancers13123089

**Published:** 2021-06-21

**Authors:** Chuan Zhang, Mandy Berndt-Paetz, Jochen Neuhaus

**Affiliations:** 1 Department of Urology, University of Leipzig, 04109 Leipzig, Germany; Chan.Zhang@medizin.uni-leipzig.de (C.Z.); Mandy.Berndt@medizin.uni-leipzig.de (M.B.-P.); 2 Department of Urology, Chengdu Fifth People’s Hospital Affiliated to the Chengdu University of Traditional Chinese Medicine, Chengdu 611130, China

**Keywords:** Notch pathway, bioinformatics analysis, bladder cancer, prognosis, immune system modulation

## Abstract

**Simple Summary:**

The Notch pathway is important in embryology and numerous tumor diseases. However, its role in bladder cancer (BCa) has not been deeply investigated thus far. Gene expression data are available for BCa, and bioinformatics analysis can provide insights into a possible role of the Notch pathway in BCa development and prognosis. Using this information can help in better understanding the origin of BCa, finding novel biomarkers for prediction of disease progression, and potentially opening new avenues to improved treatment. Our analysis identified the Notch receptors NOTCH2/3 and their ligand DLL4 as potential drivers of BCa by direct interaction with basic cell functions and indirect by modulating the immune response.

**Abstract:**

Background: A hallmark of Notch signaling is its variable role in tumor biology, ranging from tumor-suppressive to oncogenic effects. Until now, the mechanisms and functions of Notch pathways in bladder cancer (BCa) are still unclear. Methods: We used publicly available data from the GTEx and TCGA-BLCA databases to explore the role of the canonical Notch pathways in BCa on the basis of the RNA expression levels of Notch receptors, ligands, and downstream genes. For statistical analyses of cancer and non-cancerous samples, we used R software packages and public databases/webservers. Results: We found differential expression between control and BCa samples for all Notch receptors (NOTCH1, 2, 3, 4), the delta-like Notch ligands (DLL1, 3, 4), and the typical downstream gene hairy and enhancer of split 1 (HES1). NOTCH2/3 and DLL4 can significantly differentiate non-cancerous samples from cancers and were broadly altered in subgroups. High expression levels of NOTCH2/3 receptors correlated with worse overall survival (OS) and shorter disease-free survival (DFS). However, at long-term (>8 years) follow-up, NOTCH2 expression was associated with a better OS and DFS. Furthermore, the cases with the high levels of DLL4 were associated with worse OS but improved DFS. Pathway network analysis revealed that NOTCH2/3 in particular correlated with cell cycle, epithelial–mesenchymal transition (EMT), numbers of lymphocyte subtypes, and modulation of the immune system. Conclusions: NOTCH2/3 and DLL4 are potential drivers of Notch signaling in BCa, indicating that Notch and associated pathways play an essential role in the progression and prognosis of BCa through directly modulating immune cells or through interaction with cell cycle and EMT.

## 1. Introduction

The Notch signaling pathway is a highly conserved ligand-receptor signaling pathway involved in various aspects of cancer biology that plays an essential role in cancer stemness, angiogenesis, epithelial–mesenchymal transition (EMT), tumor immunity, and drug resistance [1,2,3]. In recent years, Notch pathways have been highlighted in the progression and prognosis of bladder cancer (BCa) [4]. However, the literature results are quite different.

Hayashi et al. described NOTCH2 as an oncogene that is overexpressed in BCa with a high copy number gain, promoting the progression and invasion of BCa [5]. In contrast, Greife et al. found that NOTCH2 was significantly downregulated in a set of invasive BCa samples at the mRNA level [6]. Maraver et al. supported the results from Greife and suggested that NOTCH2 serves as a tumor suppressor, indicating that the loss of NOTCH2 activity seems to favor the EMT, leading to more aggressive BCa [7]. Rampias et al. focused on NOTCH1 and described the fact that NOTCH1 is a tumor suppressor in BCa. Additionally, with less evidence, they also supposed a tumor-suppressive role for NOTCH2 and NOTCH3 [4,8]. However, Zhang et al. reported that NOTCH3 was significantly overexpressed in BCa and associated with a poor prognosis [9]. Overall, in the literature there is a significant inconsistency of expression levels and dichotomous roles of the Notch pathway in BCa. Notably, Notch ligands may also play an essential role in the progression and development of BCa [4]. Overall, the mechanisms and functions of Notch pathways in BCa are still unclear, and the mechanisms of action remain elusive. 

Cancer results from the co-evolution of malignant cells and the tumor microenvironment (TME) [10]. The progression, development, metastasis, invasion, and resistance to therapy are modulated by bidirectional interactions between the TME and cancer cells [11]. The immune infiltrates are a critical part of the TME and play a vital role in bladder cancer progression [12,13]. Therefore, knowing genes modulating the immune response is critical to learn more about how cancer cells evade or how they suppress anti-tumorigenic immune responses. To date, Notch signaling in the tumor microenvironment has been widely reported in various cancers [10]. However, the mechanisms and correlation between the Notch members and immune responses in BCa have rarely been investigated. 

Various well-known cancer-related pathways are involved in carcinogenesis, such as Wnt, Hedgehog, hypoxia, and TGF/BMP pathways, which potentially affect Notch signaling [14]. Furthermore, some pathways have been shown to play a critical role in the BCa, e.g., tuberous sclerosis complex–mammalian target of rapamycin (TSC-mTOR) pathway (TSC/mTOR) [15], receptor tyrosine kinase signaling (RTK) [16], Ras/mitogen activated protein kinase pathway (RAS/MAPK) [17], epithelial–mesenchymal transition (EMT) [18], DNA damage response [18], cell cycle [19], and apoptosis pathways [20,21]. Whether the crosstalk with the well-known cancer-related pathways can affect the dichotomous function of Notch signaling in BCa is still unclear.

**Figure 1:**
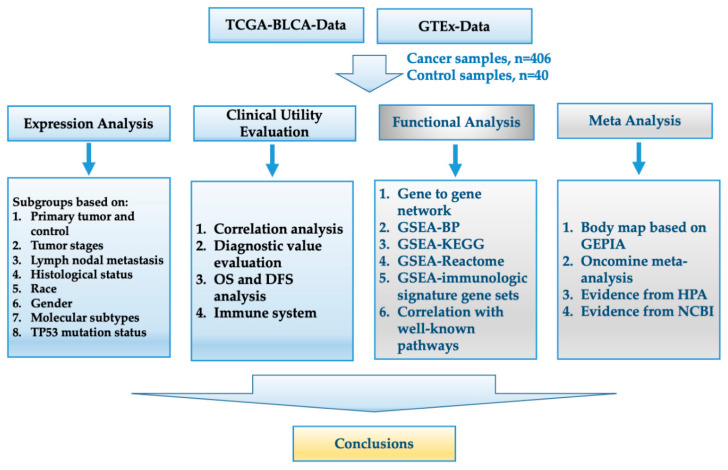
Study design. The workflow of the analysis steps. TCGA: The Cancer Genome Atlas; BLCA: bladder cancer; GTEx: the genotype-tissue expression; OS: overall survival; DFS: disease-free survival; GSEA: gene set enrichment analysis; BP: biological process; KEGG: Kyoto Encyclopedia of Genes and Genomes; GEPIA: gene expression profiling interactive analysis; HPA: human protein atlas; NCBI: National Center for Biotechnology Information.

Taken together, on the basis of the mRNA expression levels in BCa, the most interesting actors in Notch signaling are (i) the Notch receptors: NOTCH1, NOTCH2, NOTCH3, NOTCH4; (ii) the serrate-like, canonical Notch ligands Jagged 1 (JAG1) and Jagged 1 (JAG2), as well as the Delta-like canonical Notch ligand 1 (DLL1), DLL3, DLL4; (iii) the typical downstream genes: hairy and enhancer of split 1 (HES1) and hairy/enhancer-of-split-related with YRPW motif protein 1 (HEY1). The present study, through comprehensive bioinformatics analysis, tries to elucidate the molecular mechanisms and biological processes of Notch signaling in BCa, including its possible effects on the tumor immune response and the correlation with well-known cancer-related pathways (Figure 1). 

## 2. Materials and Methods

### 2.1. Acquisition of Data

Target gene expression data of normal bladder samples were downloaded from the GTEx (https://gtexportal.org/home/datasets; accessed on 20 November 2020) database [22,23]. The clinical datasets and target gene expression for non-cancerous samples and bladder cancer samples (TCGA-BLCA) were downloaded from TCGA (https://portal.gdc.cancer.gov/; accessed on 18 September 2020) [24,25]. HTseq-count files were used to tabulate the number of uniquely mapping reads for each gene. We used statistical software R (Version 4.0.2. https://www.r-project.org/; accessed on 20 June 2020) with R studio (Version 1.2.5042) [26] and “DESeq2” R package to normalize the data and performed batch correction of the gene expression between sample in order to make features comparable. All the data used in this study are open for research, and we used the data following the guidelines in the appropriate Data Use Certification Agreement.

### 2.2. Analysis of Expression Levels in Different Subgroups

Cell type-specific expression levels were not available in the TCGA and GTEX datasets; however, we could infer the relative abundance of tumor-infiltrating lymphocytes (TILS) by using gene set variation analysis (GSVA) based on gene expression profile retrieved from the tumor tissue-TCGA-BLCA, allowing lymphocyte subtype analysis.

We analyzed the following subgroups: (i) primary tumor, non-cancerous samples; (ii) tumor stage: stage I, stage II, stage III, stage IV [27,28,29,30,31,32,33,34,35]; (iii) lymph nodal metastasis: N0 (no regional lymph node metastasis), N1 (metastasis in 1 to 3 axillary lymph nodes), N2 (metastasis in 4 to 9 axillary lymph nodes), N3 (metastasis in 10 or more axillary lymph nodes) [33]; (iv) histological status: papillary tumor (PT), non-papillary tumor (NPT) [36,37]; (v) race: Asian, Black or African American, and Caucasian [38]; (vi) gender: female vs. male patients [38,39]; (vii) molecular subtypes: neuronal (NET), basal (BT), basal squamous (BST), luminal (LT), luminal infiltrated (LIT), luminal papillary (LPT) [36,40]; (viii) mutated TP53, TP53 non-mutate [41].

We used the statistical software R studio, including the packages of “Bioconductor”, “tidyr”, “complexHeatmap, “RColorBrewer”, “Biocmanager”, “circlize”, and “ggplot2” for statistical analysis and results visualization. UALCAN (http://ualcan.path.uab.edu; accessed on 20 December 2020) [42,43,44] was used as an aided analysis tool to analyze the expression levels of the target genes across different subgroups on the basis of molecular subtypes and TP53 mutation status. We excluded subgroups with sample sizes < 7; *p*-value < 0.05 was considered statistically significant.

### 2.3. Correlation Analysis and Evaluation of the Diagnostic Value

For correlation analysis and correlation matrix visualization, we used the statistical software R studio with the packages “performanceAnalytics”, “Hmisc”, “pROC”, and “corrplot”. We analyzed the correlation between different target genes. In addition, according to the tumor and immune system interaction database (TISIDB; http://cis.hku.hk/TISIDB; accessed on 18 March 2021) [45,46], correlations between the Notch members (Notch receptors and Notch ligands) with lymphocytes and the relationship with immunomodulators in BCa were analyzed. Criteria of correlation co-efficients were defined as follows: 0.00–0.19 (very weak), 0.20–0.39 (weak), 0.40–0.59 (moderate), 0.60–0.79 (strong), and 0.80–1.0 (very strong). A *p*-value < 0.05 was considered statistically significant [43]. We used receiver operating characteristic (ROC) curve analysis to evaluate the diagnostic value of the genes in question. Additionally, in order to identify the critical factors of Notch signaling, we used orthogonal partial least squares discriminant analysis (OPLS-DA; multivariate statistical analysis by R packages “ropls”). For quality criterion, we chose R^2^X > 0.4 in the PCA model, and R^2^Y (goodness of fit parameter) and Q^2^ (predictive ability parameter) > 0.5 in OPLS-DA [47,48].

### 2.4. Overall Survival (OS) and Disease-Free Survival (DFS) Analyses

Clinical data were downloaded from TCGA-BLCA and analyzed with R studio (packages: “ComplexHeatmap”, “clusterProfiler”, “survival”, “survMisc”, “survminer”, “RColorBrewer”). Kaplan–Meier estimates of OS and DFS were performed to evaluate the prognostic value of target genes. We used Cox regression to find independent factors, landmark analysis, and time-dependent covariates to evaluate the prognostic value in case of crossing over between groups. We also used GSCALite (http://bioinfo.life.hust.edu.cn/web/GSCALite/, accessed on 20 March 2021) [49,50], a web server for gene set cancer analysis, to estimate whether DNA methylation of Notch member genes had effects on the overall survival. A *p*-value < 0.05 was considered statistically significant. Data on cancer-specific survival are not available in the TCGA-BLCA dataset.

### 2.5. Gene–Gene Interaction (PPI) Network Analysis and Gene Set Enrichment Analysis (GSEA)

We used GeneMANIA (http://genemania.org/co-expression genes; accessed on 12 November 2020) [51,52] to construct the gene–gene interaction network (GGI) and GSEA software (Version 4.0.3) [53] for the functional analysis of the BCa and non-cancerous samples. We included biological process (BP) of Gene Ontology (GO) term enrichment analysis, canonical pathway analysis based on Kyoto Encyclopedia of Genes and Genomes (KEGG) pathway, REACTOME database analysis, and immunologic signature gene set analysis. Analysis results of GSEA, satisfying the threshold of normalized enrichment score |NES| > 1, nominal *p*-value (NOM *p*-value) < 0.05, and a false discovery rate (FDR) q-value < 0.10 were considered statistically significant. 

Additionally, we also utilized the web server GSCALite (http://bioinfo.life.hust.edu.cn/web/GSCALite/, accessed on 20 March 2021) [49,50] to evaluate the relationship between target genes and other well-known BCa-related pathways, including tuberous sclerosis complex–mammalian target of rapamycin (TSC-mTOR) pathway (TSC/mTOR) [15], receptor tyrosine kinase signaling (RTK) [16], Ras/mitogen-activated protein kinase pathway (RAS/MAPK) [17], phosphatidylinositol 3-kinase and the serine/threonine kinase pathway (PI3K/AKT) [54], hormone estrogen receptor (ER) [55], hormone androgen receptor (AR) [56], epithelial–mesenchymal transition (EMT) [18], DNA damage response [18], cell cycle [19], and apoptosis pathways [20,21]. The percentage of BCa cases in which those pathways may be activated or inhibited by Notch genes were also assessed. 

### 2.6. Body Maps of the Target Genes and Oncomine Analysis

The expression levels of Notch-related genes were body mapped for various cancers and normal tissues using Gene Expression Profiling Interactive Analysis (GEPIA, http://gepia.cancer-pku.cn; accessed on 12 November 2020) [57,58]. Additionally, we used Oncomine (https://www.oncomine.org; accessed on 13 November 2020) [59,60] for metanalysis of the target genes in previous studies. Immunochemistry (IHC) stainings of the proteins coded by the target genes in BCa and control tissues were accessed from the Human Protein Atlas (HPA, http://www/proteinatlas.org/; accessed on 13 December 2020) [61,62]. A *p*-value < 0.05 was considered statistically significant.

### 2.7. Other Statistical Analyses

All statistical analyses were performed using R statistical software. Univariate analysis was performed using ANOVA, *t*-test, Wilcoxon test, Tukey’s HSD, and permutation tests. The false discovery rate (FDR) was used to conceptualize the rate of errors in null hypothesis testing, and Bonferroni/familywise error rate (FWER) was used to adjust *p*-values. Correlation analysis was performed by Pearson test or Spearman analysis. Multivariate analyses were performed using the principal component analysis (PCA) and orthogonal partial least squares discriminant analysis (OPLS-DA) model. Receiver operating characteristic (ROC) curve analysis was performed to evaluate the diagnostic value. Kaplan–Meier estimates of OS and DFS, landmark analysis, and time-dependent covariates were used to evaluate the prognostic value of target genes. We used Cox regression for survival analysis to find the vital factors. A *p*-value < 0.05/adjusted *p*-value < 0.05 were considered statistically significant.

## 3. Results

Four hundred and six tumor samples (*n* = 406) and 19 non-cancerous samples were available from TCGA-BLCA, and 21 normal bladder samples were extracted from GTEx. The normalized data passed the quality control and kept the differences in expression levels (Figure S1). We defined non-cancerous bladder tissues from TCGA-BLCA and normal bladder samples extracted from GTEx as controls (*n* = 40) (Table S1). The demographic and patient characteristics including tumor classifications are shown in Table 1.

### 3.1. Gene Expression Analysis

#### 3.1.1. Gene Expression of Notch Pathway-Related Genes in BCa and Controls

**Figure 2:**
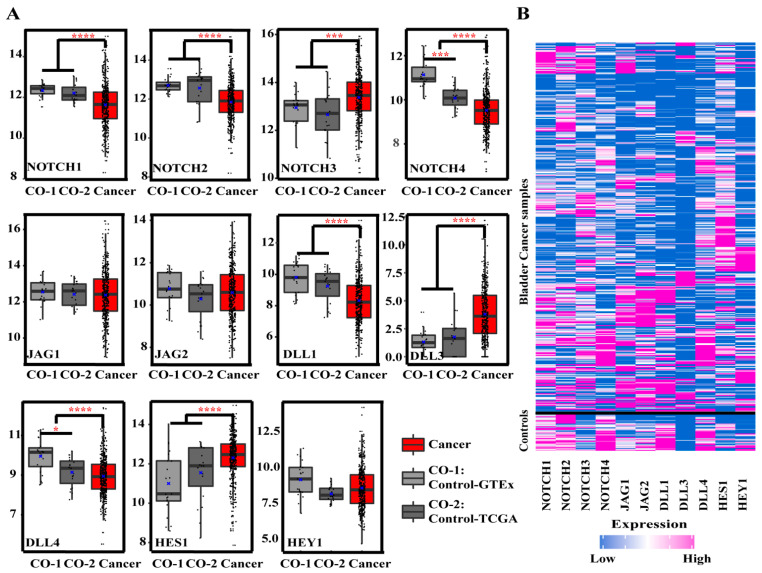
Expression levels of target genes in the control group and BCa samples. (**A**) Box plots of research gene expression levels between control samples (gray, *n* = 40) and BCa samples (red, *n* = 406). *y*-axis = Log_2_(read count value). Controls comprise normal bladder samples from GTEx (CO-1 = control-GTEx, *n* = 21) and non-cancerous bladder samples from TCGA-BLCA (CO-2 = control-TCGA, *n* = 19), and significance levels are marked as * *p*-value < 0.05, *** *p*-value < 0.001, **** *p*-value < 0.0001. (**B**) Heatmap of expression levels (color-coded). High expression marked in pink; low expression marked in blue.

Compared with controls, the expression levels of NOTCH1, NOTCH2, NOTCH4, DLL1, and DLL4 were significantly downregulated in the BCa samples. On the contrary, NOTCH3, DLL3, and HES1 were significantly overexpressed. Interestingly, in controls, NOTCH4 and DLL4 in non-cancerous samples from TCGA-BLCA were significantly lower than in normal tissues from GTEx. No significant differences were found for JAG1, JAG2, and HEY1 (Figure 2, Table 2). In the Supplementary Materials, we included a complete set of figures illustrating the expression of Notch-related genes with respect to tumor TNM stage (Figure S2), lymph node status (Figure S3), histologic type (papillary vs. non-papillary; Figure S4), and molecular subtypes (Figure S5).

#### 3.1.2. Gene Expression in Different Tumor Stages

In tumor stage I, only two samples were available. Therefore, we omitted stage I from the analysis between different tumor stages. The expression of NOTCH2 was lower in stage II (S2) than in stage III (S3) and stage IV (S4); JAG2 was higher in S2 than in S4 (Table 2).

#### 3.1.3. Gene Expression in Patients Stratified for Lymph Nodal Metastasis

Compared to controls, we found that NOTCH2 was significantly downregulated in N0, N1, and N2. NOTCH3 and HES1 were significantly upregulated in N0, N1, and N2. NOTCH4, DLL1, and DLL3 were significantly altered in a range from N0, N1, N2, to N3. Furthermore, no significant differences were found between N1, N2, and N3, and HEY1 was also not significantly changed between subgroups (Table 2). The NOTCH gene expression was up- or downregulated without correlation to lymph nodal metastasis status (data not shown).

#### 3.1.4. Gene Expression in Papillary (PT) and Non-Papillary Tumors (NPT)

Compared to control samples, NOTCH3, DLL3, and HES1 were significantly increased in PT and NPT, whereas NOTCH1, NOTCH2, NOTCH4, and DLL1 were significantly decreased in PT and NPT.

Analysis of papillary vs. non-papillary BCa showed lower expression levels of NOTCH2, JAG1, DLL1, and DLL3 in PT compared to NPT, whereas NOTCH4, DLL4, and HES1 were upregulated in PT (Table 2).

#### 3.1.5. Gene Expression in Patients Stratified for Race and Gender

We found significant differential expression of NOTCH2 and DLL4 between Asians (ASI, *n* = 43), Caucasians (CAU, *n* = 323), and Black or African Americans (AFA, *n* = 23; Table 2). NOTCH1, JAG1, and JAG2 were significantly upregulated in AFA compared to CAU, while NOTCH4 was significantly upregulated in ASI compared to AFA. No significant differences were found in the expression levels of NOTCH3, DLL1, DLL3, and HEY1.

The cohort included *n* = 299 male and *n* = 107 female patients (gender ratio of 3:1). The gender ratio was 2:1 (*n* = 14 male; *n* = 5 female) in the control group (Table S1). Only JAG2 and DLL3 showed gender specific differential expression with higher expression levels in female patients (Table 2).

#### 3.1.6. Gene Expression in Molecular Subtypes

We investigated the gene expression in neuronal (NET), basal (BT), basal squamous (BST), luminal (LT), luminal infiltrated (LIT), and luminal papillary (LPT). Compared to non-cancerous tissue controls from the TCGA-BLCA dataset (C-TCGA), NOTCH1 was downregulated only in LT; NOTCH2 was downregulated in LT, LIT, and LPT; and NOTCH4 was downregulated in BST. In contrast, NOTCH3 was upregulated in LT, LIT, LPT, and BST (see Table 3 for a complete list of all investigated target genes).

#### 3.1.7. Gene Expression in Patients Stratified for Tumor-Suppressor TP53 Mutation

According to the TP53 mutation status, we separated the BCa samples into the cases with mutation (TP53^M^) and without mutation (TP53^WT^). Compared to C-TCGA, patients with TP53 mutation showed downregulation of NOTCH1, NOTCH2, and NOTCH4, while NOTCH3 was upregulated. Patients without mutation showed downregulated NOTCH2 and upregulation of NOTCH4 only. Comparison of the TP53 mutation status in BCa tissue revealed in TP53^WT^ higher expression of NOTCH1 and NOTCH4 but lower expression of NOTCH2 (see Table 3 for a complete list of all investigated target genes).

### 3.2. Correlation and Diagnostic Value of Notch-Related Genes, and Correlation between Notch Pathway, Lymphocyte Subtypes, and Immunomodulators

#### 3.2.1. Correlations between Notch-related genes in the BCa Cohort 

**Figure 3:**
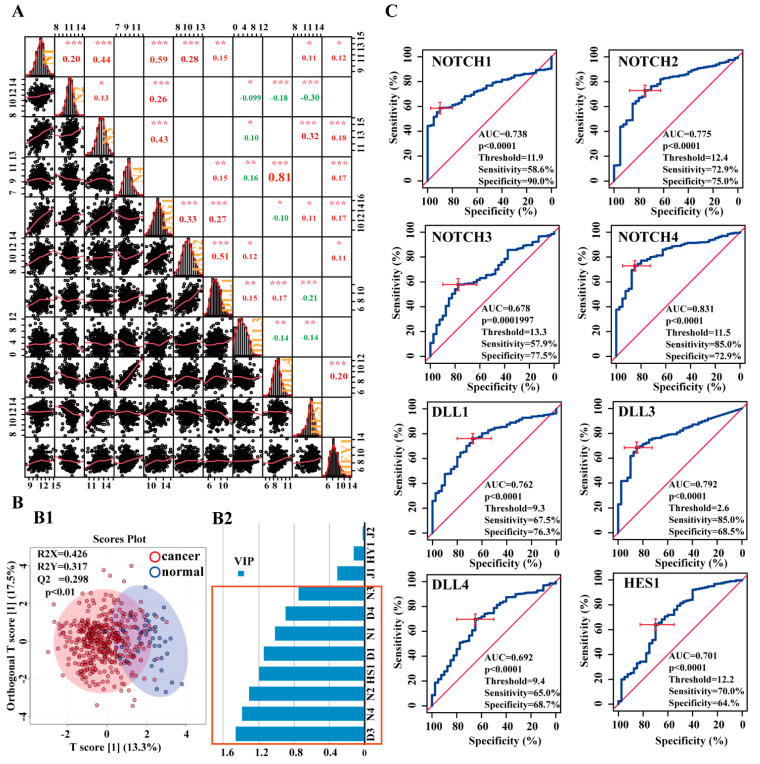
Correlations and diagnostic value of Notch pathway-related genes. (**A**) Correlation between the target genes in BCa (*n* = 406); explanation of the histogram: (i) the histogram of the kernel density estimation and distribution of each component is shown on the diagonal; (ii) on the bottom of the diagonal: the bivariate scatter plots with a fitted line are displayed; (iii) on the top of the diagonal: the value of the correlation plus the significance level as stars; each significance level is associated to a symbol: *p*-values 0.001, 0.01, 0.05 relate to symbols, ***, **, *, respectively; the number in the charts is the Pearson’s correlation coefficient (r), the positive marked in red and negative marked in green; (iv) numbers at the sides of the charts indicate the range of expression values = Log_2_(read count value). N1: NOTCH1, N2: NOTCH2, N3: NOTCH3, N4: NOTCH4, D1: DLL1, D3: DLL3, D4: DLL4, J1: JAG1, J2: JAG2, HS1: HES1, HY1: HEY1. (**B**) OPLS-DA results. (**B1**) OPLS-DA-based separation of the BCa and controls. (**B2**) VIP of each feature from OPLS-DA. (**C**) Representative ROC curves showing the diagnostic accuracy (AUC) of BCa detection on the basis of NOTCH1, NOTCH2, NOTCH3, NOTCH4, DLL1, DLL3, DLL4, and HES1; the red cross marked in (**C**) is the best threshold position/value.

Correlation analysis revealed a strong positive correlation between NOTCH4 and DLL4 (Pearson’s correlation coefficient *r* = 0.81, *p*-value < 0.001) and moderate positive correlations between NOTCH1 and JAG1 (*r* = 0.59, *p*-value < 0.001), NOTCH3 and JAG1 (*r* = 0.43, *p*-value < 0.001), and JAG2 and DLL1 (*r* = 0.51, *p*-value < 0.001) (Figure 3A). 

#### 3.2.2. Potential Diagnostic Value of Notch-Related Genes

As shown in Figure 3B1, the majority of BCa cases (*n* = 406) could be discriminated from controls (*n* = 40) by the OPLS-DA scores plot. Moreover, this OPLS-DA model met the quality criterion R^2^X > 0.4. However, the fitting of the data was R^2^Y = 0.317 (*p* < 0.01) and the predictive power was Q2 = 0.298 (*p* < 0.01). Nevertheless, the variable importance in projection (VIP) in OPLS-DA identified eight potential critical genes in descending order: DLL3, NOTCH4, NOTCH2, HES1, DLL1, NOTCH1, DLL4, and NOTCH3 (Figure 3B2).

Receiver operating characteristic (ROC) curve analysis revealed high predictive values with *p*-value ≤ 0.0001 for the Notch receptors: NOTCH1 (AUC = 0.738, sensitivity = 58.6%, specificity = 90%), NOTCH2 (AUC = 0.775, sensitivity = 72.9%, specificity = 75.0%), NOTCH3 (AUC = 0.678, sensitivity = 57.9%, specificity = 77.5%), and NOTCH4 (AUC = 0.831, sensitivity = 72.9%, specificity = 85.0%) (Figure 3C). 

The Notch ligands with the highest predictive abilities (*p*-value < 0.0001; Figure 3C) were DLL1 (AUC = 0.762, sensitivity = 76.3%, specificity = 67.5%), DLL3 (AUC = 0.792, sensitivity = 68.5%, specificity = 85.0%), and DLL4 (AUC = 0.692, sensitivity = 68.7%, specificity = 65.0%). The downstream gene HES1 had an AUC = 0.701 (sensitivity = 64.0%, specificity = 70.0%). The AUCs of JAG1, JAG2, and HEY1 were lower than 0.70 and not significant (*p*-value > 0.05; Figure S6). The combination of various biomarkers did not result in significantly higher predictive values (Table S2). 

#### 3.2.3. Notch Pathway Correlated with Lymphocyte Subtypes and Immunomodulating Genes

**Figure 4:**
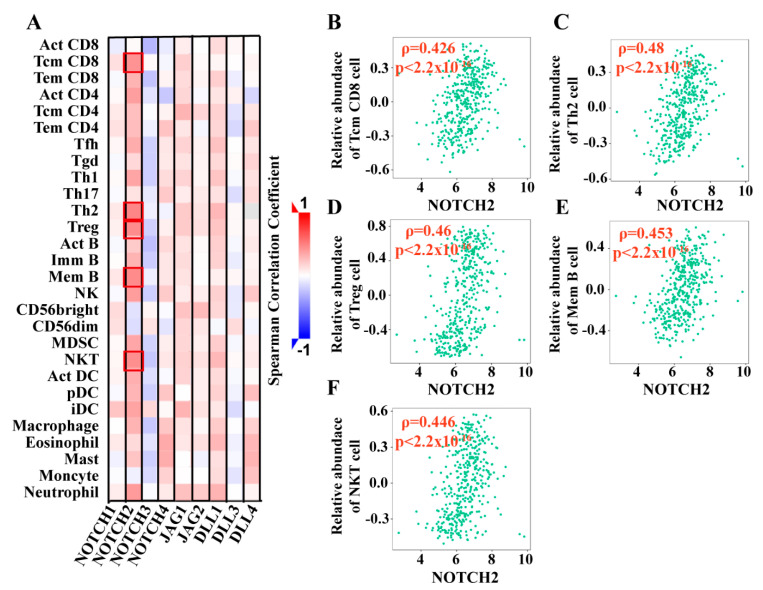
Correlation between Notch factors and lymphocytes. (**A**) The heatmap of correlation between the target genes and lymphocytes in BCa (*n* = 408). (i) the value of Spearman correlation coefficient is color-coded; high value is marked in red; low value was marked in blue. (ii) The most significant results are marked with a red frame. (**B**–**F**) Representative moderate positive correlation of NOTCH2 with Tcm CD8 cells (**B**), Th2 cells (**C**), Treg cells (**D**), Mem B cells (**E**), and NKT cells (**F**) in BCa; *p*-value *(p)*. *x*-axis = expression level of target gene, *y*-axis = relative abundance of lymphocytes, scatter plots show curved patterns of the samples. TISIDB (http://cis.hku.hk/TISIDB/browse, accessed on 18 March 2021) [46].

Correlation analysis was performed between the Notch receptors and ligands and the infiltration level/relative abundance of lymphocytes in BCa. NOTCH2 showed the strongest, mostly positive, correlation with lymphocytes. In contrast, NOTCH3 was negatively correlated with the majority of lymphocytes (Figure 4A). We focused on relationships with *ρ* > 0.4 and *p*-value < 0.05. 

NOTCH2 showed a moderate positive correlation with CD8 positive central memory T cells (Tcm CD8 cells, *ρ* = 0.426, *p*-value < 2.2 × 10^−16^) (Figure 4B), type 2 T helper cells (Th2 cells, spearman correlation coefficient; *ρ* = 0.48, *p*-value < 2.2 × 10^−16^; Figure 4C), regulatory T cell (Treg cells, *ρ* = 0.453, *p*-value < 2.2 × 10^−16^; Figure 4D), memory B cells (Mem B cells, *ρ* = 0.46, *p*-value < 2.2 × 10^−16^; Figure 4E), and natural killer T cells (NKT cells, *ρ* = 0.446, *p*-value < 2.2 × 10^−16^; Figure 4F). The other target genes showed no or only weak correlations with lymphocytes (Figure 4A, Table S3).

**Figure 5:**
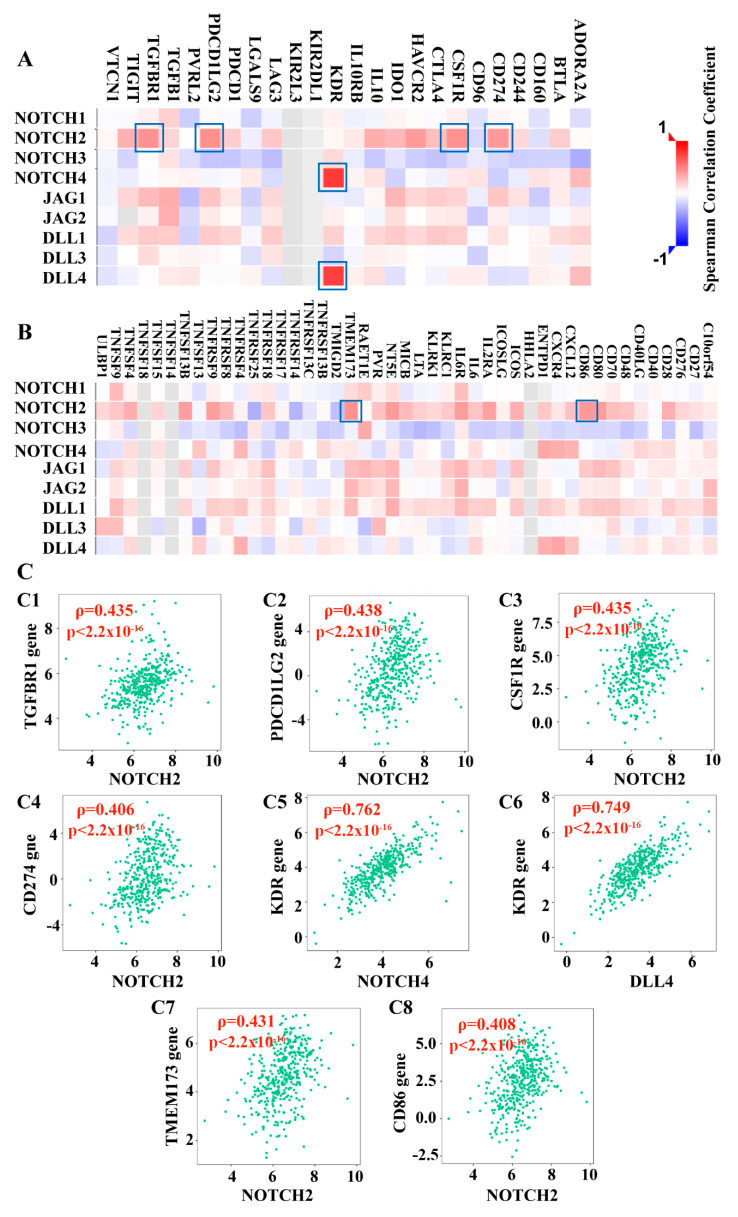
Relationship of the Notch pathway with the gene expression of immunomodulators in BCa. (**A**) The heatmap of the correlation with immunoinhibitors (*n* = 408). (**B**) The heatmap of the correlation with immunostimulators (*n* = 408). (i) The value of Spearman correlation coefficient is color-coded; high value is marked in red, low value is marked in blue. (ii) The most significant and representative results are marked with blue frames. (**C**) The representative positive correlations between Notch factors and various immunomodulators in BCa: NOTCH2 with TGFBR1 (**C1**), NOTCH2 with PDCD1LG2 (**C2**), NOTCH2 with CSF1R (**C3**), NOTCH2 with CD274 (**C4**), NOTCH4 with KDR (**C5**), DLL4 with KDR (C6), NOTCH2 with TMEM173 (**C7**), and NOTCH2 with CD86 (**C8**). *p*-value (*p*). *x*-axis = expression level of target gene, *y*-axis = expression level of immunomodulator gene; scatter plots show curved patterns of the samples. TISIDB (http://cis.hku.hk/TISIDB/browse, accessed on 18 March 2021) [46].

Immunomodulators can be classified into immunoinhibitors and immunostimulators [63]. NOTCH2 seems to be the most critical member of Notch receptors showing a positive correlation with immunoinhibitors, namely, (i) transforming growth factor beta receptor 1 (TGFBR1, *ρ* = 0.435, *p*-value < 2.2 × 10^−16^; Figure 5C1), (ii) programmed cell death 1 ligand 2 (PDCD1LG2, *ρ* = 0.406, *p*-value < 2.2 × 10^−16^; Figure 5C2), (iii) macrophage colony stimulating factor I receptor precursor (CSF1R, *ρ* = 0.435, *p*-value < 2.2 × 10^−16^; Figure 5C3), and (iv) NOTCH2 with programmed cell death 1 ligand 1 (CD274, *ρ* = 0.406, *p*-value < 2.2 × 10^−16^; Figure 5C4). 

In contrast, NOTCH3 showed significantly negative correlations with the majority of immunoinhibitors, although the correlation coefficients were comparatively low (*ρ* < 0.4, *p*-value < 0.05; Figure 5A). 

Furthermore, we found strong positive correlations between NOTCH4 and DLL4 in terms of the vascular endothelial growth factor receptor 2 (KDR, *ρ* = 0.762, *p*-value < 2.2 × 10^−16^, Figure 5C5; *ρ* = 0.749, *p*-value < 2.2 × 10^−16^, Figure 5C6). The complete dataset is presented in Table S4. 

In addition, we also found a moderate correlation between NOTCH2 and immunostimulators, including a positive correlation with stimulator of interferon genes protein (TMEM173 = STING1, *ρ* = 0.431, *p*-value < 2.2 × 10^−16^; Figure 5C7) and T-lymphocyte activation antigen CD86 (CD86, *ρ* = 0.408, *p*-value < 2.2 × 10^−16^; Figure 5C8). The detailed information about the correlations between Notch factors with immunostimulators are collected in Figure 5B and Table S5.

### 3.3. Overall Survival (OS) and Disease-Free Survival (DFS) Analyses

#### 3.3.1. Dependency of Overall Survival (OS) on the Expression Levels of Target Genes

**Figure 6:**
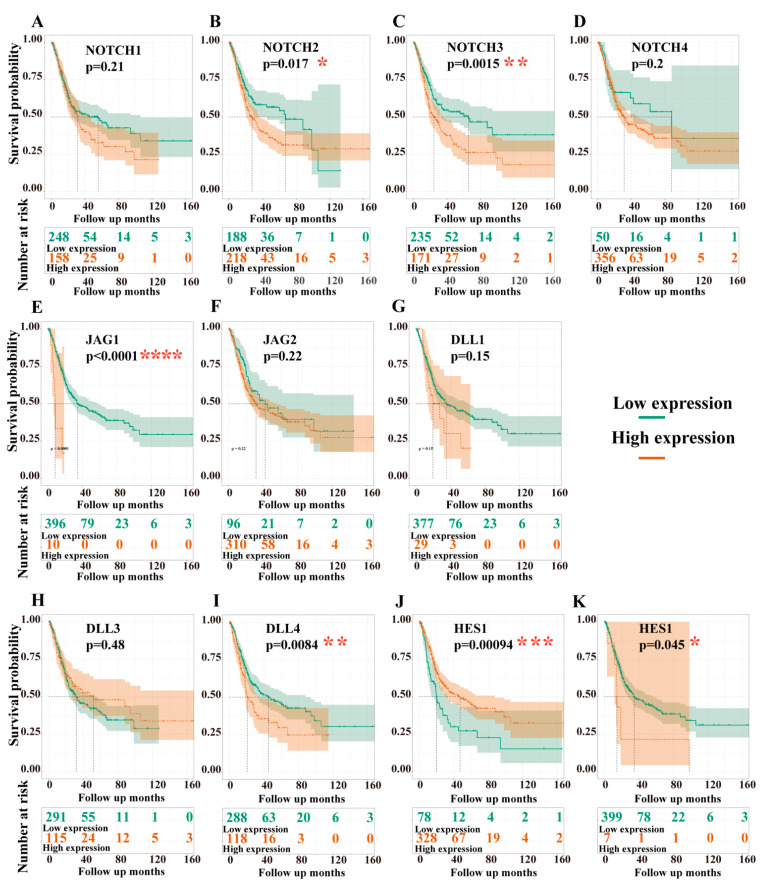
Overall survival analyses of the target genes. NOTCH1 (**A**), NOTCH2 (**B**), NOTCH3 (**C**), NOTCH4 (**D**), JAG1 (**E**), JAG2 (**F**), DLL1 (**G**), DLL3 (**H**), DLL4 (**I**), HES1 (**J**), and HEY1 (**K**); data from TCGA-BLCA, *n* = 406; dead, *n* = 179; alive, *n* = 227; see Table S6 for threshold (cut-off value) used to define the high and low levels of the expression. *y*-axis = overall survival probability, *x*-axis = follow up in days, high expression (orange line), low expression (green line); the number at risk in high- and low-expression groups are listed in the tables below the survival curve; significance levels are marked as * *p*-value < 0.05, ** *p*-value < 0.01, *** *p*-value < 0.001, **** *p*-value < 0.0001.

Kaplan–Meier estimates showed that high expression levels of NOTCH3, JAG1, DLL4, and HEY1 significantly correlated with poor OS. In contrast, high expression of HES1 was associated with prolonged survival (Figure 6). Interestingly, we found a crossing in the survival curves of NOTCH2 (Figure 6B). Subsequently, the landmark analysis revealed that high expression of NOTCH2 seems to be a risk factor in the early phase of follow-up. However, after 100 months (8.2 years, *n* = 12; high expression: *n* = 10; low expression: *n* = 2), NOTCH2 resembled an advantageous factor, with high expression being associated with a better prognosis (Figure S7A). Cox regression for overall survival (OS) analysis revealed NOTCH3, JAG1, DLL4, and HES1 as independent predictive variables in a multivariate test (Table 4).

#### 3.3.2. Dependency of Disease-Free Survival (DFS) on the Expression Levels of Target Genes

**Figure 7:**
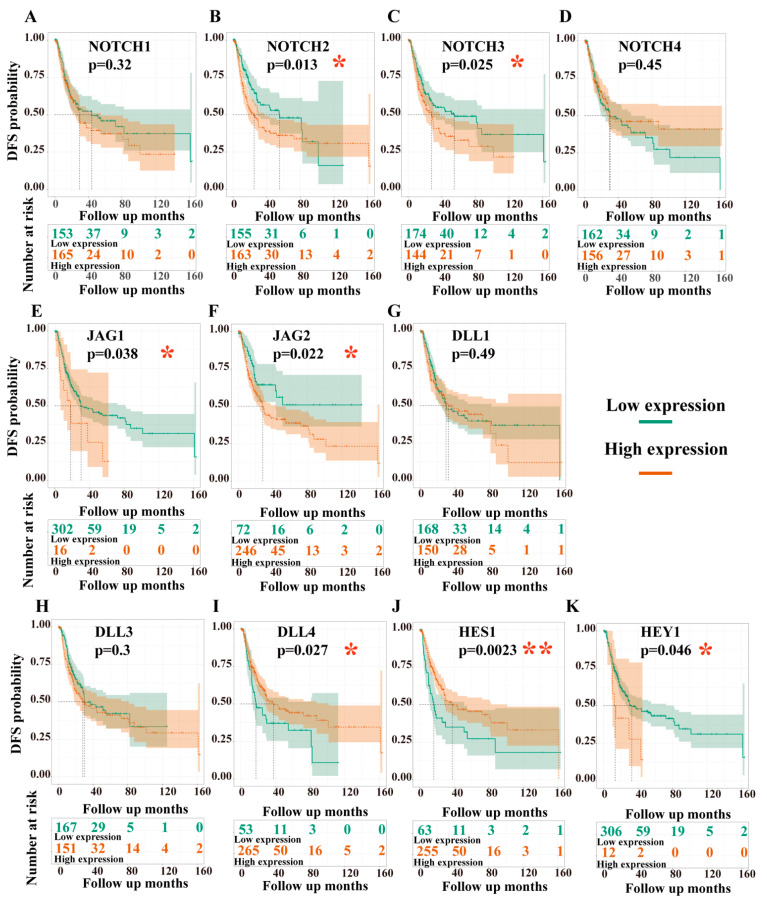
DFS analyses of the target genes. NOTCH1 (**A**), NOTCH2 (**B**), NOTCH3 (**C**), NOTCH4 (**D**), JAG1 (**E**), JAG2 (**F**), DLL1 (**G**), DLL3 (**H**), DLL4 (**I**), HES1 (**J**), and HEY1 (**K**). Data from TCGA-BLCA, *n* = 406; recurrence, *n*= 141; no recurrence, *n* = 177. The threshold (cut-off value) used to define the high or low levels of the expression groups are listed in Table S6. *y*-axis = DFS probability, *x*-axis = follow up days; high expression is marked with an orange line, low expression is marked with a green line. The number at risk in high- and low-expression groups are listed in the tables below the survival curve; significance levels are marked as * *p*-value < 0.05, ** *p*-value < 0.01.

Kaplan–Meier estimates of DFS results showed that patients with high expression of NOTCH3, JAG1, JAG2, and HEY1 had a shorter DFS than those with low expression levels. By contrast, high expression of DLL4 and HES1 was associated with better DFS (Figure 7). Intriguingly, we also found a crossing in the disease-free survival curves of NOTCH2. Landmark analysis showed that DFS was greater with higher NOTCH2 expression at up to 90 months (7.4 years, *n* = 29; high level, *n* = 17; low level, *n* = 12); thereafter, low expression of NOTCH2 was associated with greater DFS (Figure S7B). Cox regression for DFS analysis revealed NOTCH3, JAG2, and HES1 as independent variables in multivariate testing (Table 5).

#### 3.3.3. Combinations of the Independent Factors Correlated with OS and DFS, While Methylation of Notch Factors Did Not

Notch ligand binding leads to proteolysis of the Notch receptor and activation of distinct target programs, which can induce different cell fates [5,64]. Consequently, the combination of certain Notch receptors and particular ligands may be most relevant, and therefore we analyzed the correlation of several combinations of Notch members with OS and DFS on the basis of COX regression results and linear fitting models (risk score = coefficient1 * gene expression 1 +...+ coefficient N * gene expression N) [65].

We found significantly prolonged OS to be associated with low values of four combinations: (i) NOTCH3 + JAG1 (cut-off = 23.7, *p*-value = 0.047), (ii) NOTCH3 + DLL4 (cut-off = 15.7, *p*-value = 0.036), (iii) NOTCH3 + JAG1 + HES1 (cut-off = 15.6, *p*-value = 0.024), and (iv) NOTCH3 + DLL4 + HES1 (cut-off = 8.5, *p*-value = 0.0075) (Figure S8). 

Additionally, low values of NOTCH3 + JAG2 (cut-off = 10.4, *p*-value = 0.018) and NOTCH3 + JAG2 + HES1 (cut-off = 4.1, *p*-value = 0.015) were associated with prolonged DFS (Figure S8).

However, we did not find a significant influence of methylation status of the target genes on OS or DFS (data not shown).

### 3.4. Gene Networks and Gene Set Enrichment Analysis (GSEA)

#### 3.4.1. Gene Network Analysis

**Figure 8:**
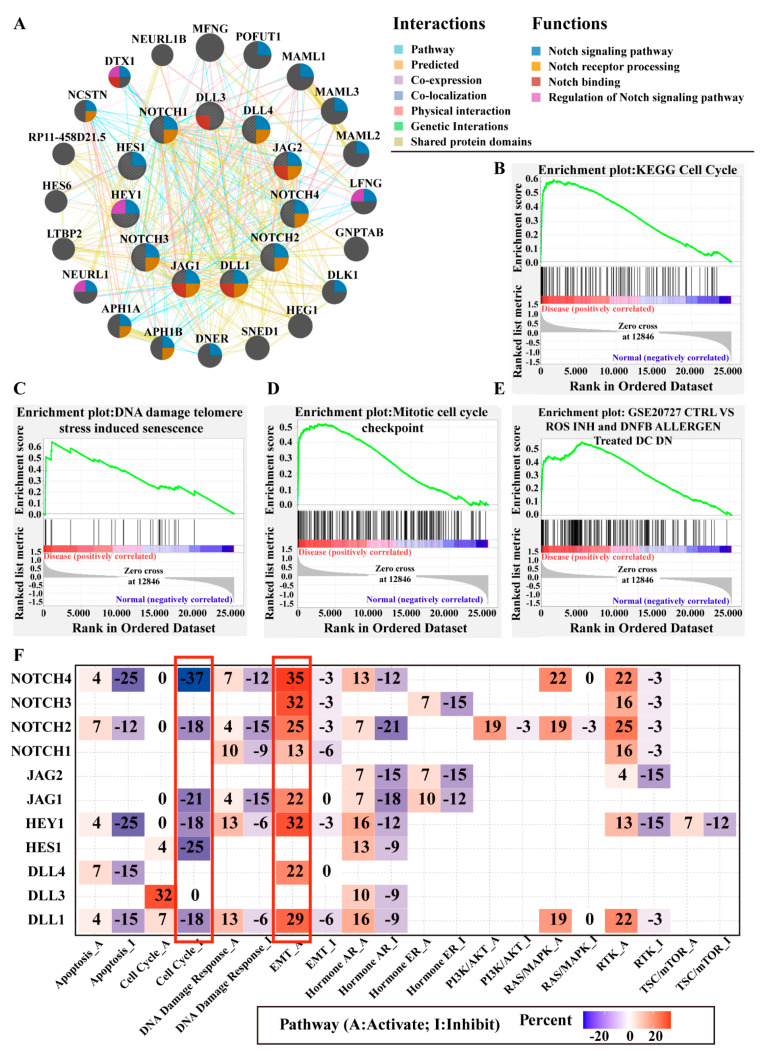
Interaction network of frequently interacting genes and the canonical pathway analysis using GSEA. (**A**) Gene to gene interaction network with frequently interacting genes (GeneMANIA, accessed on Nov.12. 2020): (i) the node size represents the strength of interaction; (ii) color of lines represents the types of interaction between genes (color code in Interactions legend); (iii) colored node circle sections represent the functions of respective genes (color code in Functions legend). (**B**) The most significant canonical pathways in terms of the KEGG database. (**C**) The most significant canonical pathways in terms of Reactome pathway database. (**D**) The most significant biological process according to Gene Ontology. (**E**) The most significant immunologic signature gene sets; NES = normalized enrichment score. (**F**) The relationship between Notch factors with well-known cancer-related signaling pathways; _A (activating); _I (inhibiting); values indicate the percentage of BCa cases in the associated pathway; positive values (activating, red), negative values (inactivating, blue); empty values = no significant correlation was detected; red frame indicates the two most relevant pathways. For full gene names and description of “GSE20727 CTRL vs. ROS INH and DNFB allergen-treated DC DN”, please see list of abbreviations (File S1).

We used GeneMANIA (http://genemania.org/, accessed on 12 November 2020) to construct the gene–gene interaction network [51,52]. Besides Notch factors, the network included another 20 potentially frequently interacting genes, and 435 links (interactions) were found (Figure 8A). The links included shared protein domains (45.98%), physical interactions (25.26%), pathway interactions (13.01%), genetic interactions (5.76%), and predicted interactions, based on gene neighborhood, gene co-occurrence, similar enzymatic function, same pathways and other factors [52] (4.95%), co-expression (4.81%), and co-localization (0.23%) (Table S7). 

The functional analysis of the network describes the roles of genes in Notch-associated pathways and their functions. In Figure 8A, we indicate four major functions as colored circle sections. Overall, we found 23 genes directly related to the Notch signaling pathway (blue): 11 of them (NOTCH1-4, DLL1, DLL4, JAG1, JAG2, APH1A, APH1B, NCSTN) were involved in Notch receptor processing (orange), 5 of them (DTX1, DLL3, DLL1, JAG1, JAG2) in Notch binding (red), and 4 of them (DTX1, LENG, NEURL1, HEY1) in the regulation of the Notch signaling pathway (pink). The functions overlapped in part (Figure 8A).

Additionally, the results also revealed some significant and interesting interactions with other functions including stem cell development, cell fate determination, and immune system development. Further information regarding results of the gene network analysis of function is supplied in Table S8.

#### 3.4.2. Gene Set Enrichment Analysis (GSEA)

In GSEA, the canonical pathway analysis is based on the KEGG database [66]. When the gene set was put in order by NES, we found that “cell cycle” was the most significant term (NES = 2.04, *p* < 0.001) (Figure 8B), followed by “homologous recombination” (NES = 1.89, *p* < 0.0001), “base excision repair” (NES = 1.89, *p* = 0.009), “mismatch repair” (NES = 1.89, *p* = 0.002), “DNA replication” (NES = 1.79, *p* = 0.01), “oocyte meiosis” (NES = 1.76, *p* = 0.01), and “P53 signaling pathway” (NES = 1.69, *p* = 0.017). The detail information of 10 significantly enriched pathways are listed in Table S9. Ordered by NES, the canonical pathway analysis based on Reactome Pathway Knowledgebase [67] showed significant enrichment in proliferation, p53 and DNA repair reactome gene sets, for example, “DNA damage telomere stress induced senescence“ (NES = 2.22, *p* < 0.0001) (Figure 8C), “regulation of TP53 activity” (NES = 2.21, *p* < 0.0001), and “M phase” (NES = 2.21, *p* < 0.0001)”. A complete and detailed list of the significantly enriched pathways is compiled in Table S10.

The biological process ontology analysis subset of gene ontology [68] (BP subset of GO analysis) in GSEA also showed enrichment of the gene sets involved in proliferation: “GO mitotic cell cycle checkpoint” (NES = 2.21, *p* < 0.0001) (Figure 8D), “GO cell cycle checkpoint” (NES = 2.21, *p* < 0.0001), and “GO spindle assembly” (NES = 2.20, *p* < 0.0001). The other significant results are summarized in Table S11. 

In addition, GSEA revealed immunologic signature gene sets correlating to dendritic cells (DCs), CD4 T cells, and CD8 T cells, such as “GSE20727 CTRL vs. ROS INH and DNFB allergen-treated DC DN” (NES = 2.26, *p* < 0.0001; Figure 8E), “GSE17974 2H vs. 72H untreated in-vitro CD4 T cell DN”(NES = 2.23, *p* < 0.0001), and “GSE10239 Memory vs. KLRG1INT EFF CD8 T cell DN” (NES = 2.19, *p* = 0.002) (Table S12).

#### 3.4.3. Notch Pathway Interactions with Other Well-Known Cancer-Related Pathways

Using GSEA, we evaluated the relationship between the target genes and other well-known cancer-related pathways. Furthermore, we also calculated the percentage of BCa cases in which the Notch pathway was either activated or inhibited by Notch genes. Analysis revealed that the Notch pathway plays a critical role in cell cycle and EMT, as indicated by high positive (activating) or negative (inhibiting) values for NOTCH4, NOTCH2, NOTCH3, JAG1, DLL1, and DLL4 (Figure 8F). The results also indicated that the target genes can have dual roles in the same pathway, e.g., NOTCH4 predominantly activates EMT (EMT_A = 35%) but can also inactivate EMT (EMT_I = 3%). Furthermore, there was a high variability in the functional role of ligands, Notch receptors, and downstream genes with respect to the cancer-related signaling pathways analyzed (Figure 8F). 

### 3.5. Oncomine Analysis of Notch-Related Genes and Body Maps

#### 3.5.1. Meta-Analysis of Notch Factors in BCa

**Figure 9:**
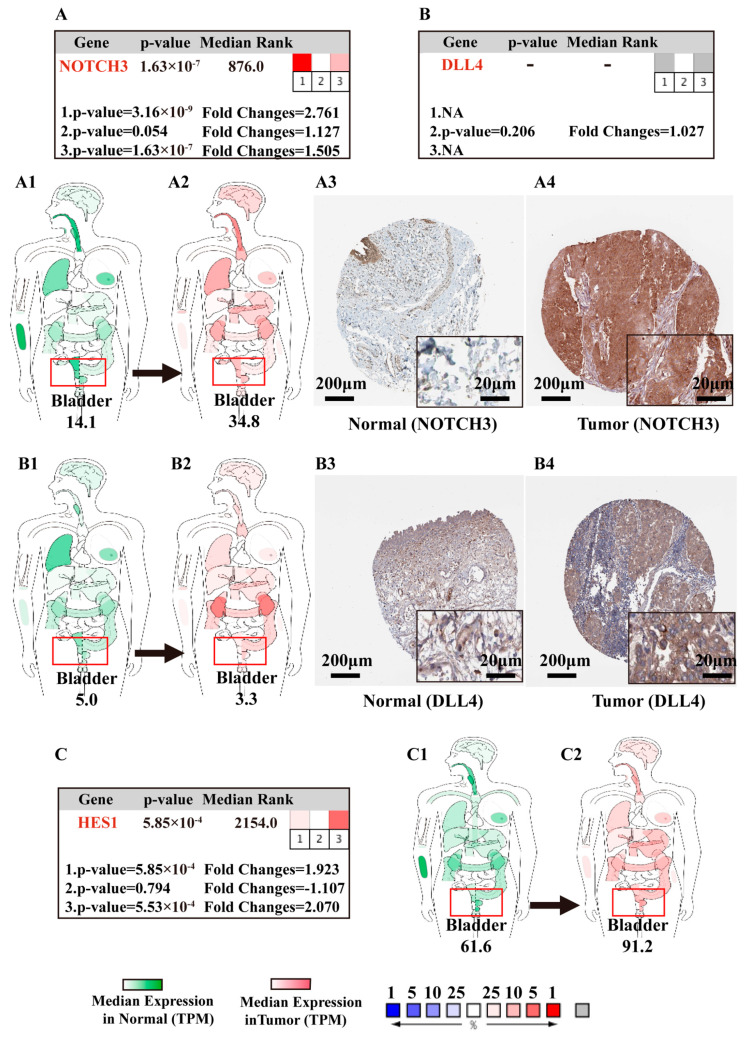
Oncomine meta-analysis of representative genes in BCa vs. non-cancerous tissue, and the body maps depicting the mRNA expression levels of the target genes. (i) Oncomine meta-analysis of NOTCH3 (**A**), DLL4 (**B**), and HES1 (**C**) in BCa vs. non-cancerous bladder tissue (https://www.oncomine.org/, accessed on 13 December 2020) [62]; heat maps of NOTCH3, DLL4, and HES1 demonstrating the gene expression in BCa samples vs. non-cancerous tissues as reported in (1) Dyrskjot et al., *Cancer Res*, 2004 [69], infiltrating bladder urothelial carcinoma (*n* = 13) vs. normal (*n* = 14); (2) Lee et al., *J Clin Oncol*, 2010 [71], infiltrating bladder urothelial carcinoma (*n* = 62) vs. normal (*n* = 10); (3) Sanchez-Carbayo et al., *J Clin Oncol*, 2006 [70], infiltrating bladder urothelial carcinoma (*n* = 72) vs. normal (*n* = 52); right hand: median rank (median rank of the gene across each of the analyses); *p*-value = *p*-value for the median-ranked analysis; color of the boxes indicates the percentile of the z-transformed expression level of the gene in the particular study; left hand: *p*-value reported in each of the studies; NA and the box being filled in gray means not measured in the study. (ii) The median expression of NOTCH3 (**A1**,**A2**), DLL4 (**B1**,**B2**), and HES1 (**C1**,**C2**) in normal tissues (green) and tumors (red) in various organs; body maps constructed on the basis of the GEPIA database (http://gepia.cancer-pku.cn, accessed on 12 November 2020) [58], transcripts per million (TPM). (iii) IHC image credit: Human Protein Atlas (https://www.proteinatlas.org/, accessed on 13 December 2020) [62]; IHC staining of NOTCH3 (**A3**) and DLL4 (**B3**) in normal tissue, NOTCH3 (**A4**) and DLL4 (**B4**) in cancerous (brown precipitates); no results on HES1 were available.

We performed an Oncomine meta-analysis based on the expression level between infiltrating bladder urothelial carcinoma vs. non-cancerous tissue (https://www.oncomine.org/, accessed on 13 December 2020) [59,60]. The meta-analysis revealed that NOTCH3 and HES1 were also significantly upregulated in BCa in previous studies [69,70] (Figure 9A,C), while the expression level of DLL4 was not significantly altered and has not been widely evaluated before [50,51,52] (Figure 9B). Interestingly, in contrast to the present study, NOTCH1, NOTCH2, and JAG1 were significantly overexpressed in BCa in Dyrskjot’s study, while the other examined genes did not show any significant difference [69]. The detail information on the other target genes is listed in Table S13.

#### 3.5.2. Body Maps of Notch Factors in Normal and BCa Patients

To visualize the distribution and to compare the expression of target genes in normal and BCa patients, we constructed the expression body maps of the genes from the Gene Expression Profiling Interactive Analysis (GEPIA) database (http://gepia.cancer-pku.cn, accessed on 12 November 2020) [57,58]. Figure 9 depicts NOTCH3, DLL4, and HES1 as representative genes of the Notch pathway. The gene expression of NOTCH3 and HES genes was upregulated in BCa 2.47 times and 1.48 times, respectively (Figure 9A1,A2,C1,C2), while DLL4 was downregulated in BCa 0.66 times (Figure 9B1,B2). Detailed information on the gene expression of all target genes is summarized in Figure S9.

#### 3.5.3. IHC of Notch-Related Proteins in Normal Bladder Tissue and BCa Samples

Immunohistochemistry (IHC) protein expression data from the Human Protein Atlas (HPA; https://www.proteinatlas.org/, accessed on 13 December 2020) [62] confirmed the expression and alteration of NOTCH3 (Figure 9A3,A4) and DLL4 proteins (Figure 9B3,B4) in BCa, No protein expression data were available for HES1, NOTCH4, DLL1, and DLL3, whereas the others can be found in Figure S9.

## 4. Discussion

### 4.1. NOTCH2/3 and DLL4 Are Potential Drivers of Notch Signaling in BCa

In recent years, great effort has been put into studying the mechanisms and prognostic and predictive values of the Notch pathway in BCa, including NOTCH1, NOTCH2, NOTCH3, NOTCH4, DLL1, DLL3, DLL4, JAG1, JAG2, HES1, and HES1. In reviewing the literature, prior studies have noted and focused on the importance of NOTCH1 and NOTCH2. However, very little of the literature has addressed the role of ligands in BCa [4]. Several previous studies have documented expression level alterations in BCa and have also described the Notch pathway’s binary action, either oncogenic or suppressive (Table 6). Nevertheless, the biological function of the Notch factors is still not fully understood in BCa.

Despite apparent contradictions among precious studies (Table 6), in line with our findings, Greife et al. also reported that HES1 was overexpressed in BCa [6]. In our study, comparing BCa samples to non-cancerous tissue, the expression levels of notch receptors NOTCH1, NOTCH2, and NOTCH4 were significantly downregulated in BCa, while NOTCH3 was overexpressed. The ligands DLL1 and DLL4 were significantly downregulated in BCa, while DLL3 was overexpressed. The expression of the downstream gene HES1 was significantly upregulated in BCa. Our results confirmed previous research (in Table 6, marked with *).

The Oncomine meta-analysis results also support our present findings. In Lee’s studies, all the target genes were detected in BCa (Table S13) [71]. Moreover, the research genes in our current study were also detected in normal bladder tissue through the RNA sequencing (RNA-seq) by the National Center for Biotechnology Information (NCBI, USA; https://www.ncbi.nlm.nih.gov/pmc/, accessed on 13 December 2020), (BioProject: PRJEB4337, *n* = 95, human; Figure S10) [79,80]. HPA data were also in line with our findings documenting the detection of NOTCH1, NOTCH2, NOTCH3, JAG1, and JAG2 by IHC in normal bladder tissue and urothelial cancer (https://www.proteinatlas.org, accessed on 13 December 2020) [61,62]. In summary, previous reports as well as our present study support the hypothesis that all Notch receptors coexist in the BCa but potentially have quite different functions [5].

Suggestive evidence from previous studies revealed that the incidence, mortality, and prognosis of BCa depend on histopathology, race, gender, and molecular and morphological features [28,29,38,81]. 

Interestingly, we did not find a significant difference in the expression of JAG1, JAG2, and HEY1 between BCa samples and non-cancerous tissue. However, in subgroup analysis based on lymph nodal, histological subtypes, race, or gender, there was a significant difference in JAG1 (PT vs. NPT (**↑**), CAU vs. AFA (**↑**), AFA vs. ASI (**↓**)), and JAG2 (C vs. N2(**↓**), CAU vs. AFA (**↑**), Male vs. Female (**↑**)), while HEY1 was not differently expressed. 

In addition, compared to non-cancerous control samples, JAG1 was significantly upregulated in BST; JAG2 was significantly upregulated in NET, BST, and LPT; and HEY1 showed significant overexpression in NET, BST, LPT, and LIT. Furthermore, JAG1, JAG2, and HEY1 were differentially expressed in BST and LT. 

Taken together, our findings support the hypothesis of biological subtypes of BCa [82,83], and we also found that NOTCH2, NOTCH3, DLL4, and HES1 expression was broadly altered in BCa subgroups (Tables S14 and S15).

Therefore, it could conceivably be hypothesized that NOTCH2, NOTCH3, and DLL4 are major factors in BCa and potential drivers of the Notch pathway. Additionally, the association of different Notch genes with molecular subtypes could promote our understanding of the biological role of the Notch pathway in BCa, thereby expanding therapeutic options, molecular diagnostics, and risk stratification [84,85,86]. Furthermore, these findings could also improve our understanding of the pathogenesis of BCa [83,84,85,86,87].

### 4.2. Differentially Expressed Notch-Related Genes Discriminate BCa and Relate to BCa Prognosis 

Hu et al. documented that Notch4 receptor protein was differentially expressed in BCa compared to adjacent non-cancerous tissue and used Notch4 as one of 10 different biomarkers to discriminate MIBC from non-cancerous tissues. [73]. However, the diagnostic value of other factors is not recognized yet. In our study, ROC analyses revealed that NOTCH1-4, DLL1, DLL3, DLL4, and HES1 were able to distinguish the patients with BCa from normal individuals (BCa, *n* = 406; control, *n* = 40) with an accuracy (AUC) ranging from 0.678 (NOTCH3) to 0.831 (NOTCH4), sensitivities from 57.9% (NOTCH3) to 76.3% (DLL1), and specificities from 65% (DLL4) to 90% (NOTCH1). The OPLS-DA model confirmed the results implicated by the ROC analyses, also identifying NOTCH1-4, DLL1, DLL3, DLL4, and HES1 as potentially predominant factors, distinguishing BCa patients from normal controls. 

Rampias et al. found that NOTCH1 alterations (mutations and/or copy number losses) were associated with poor OS, and lower expression of HEY1 and HES1 correlated with poor OS or DFS [8]. Zhang et al. pointed out that patients with high expression of NOTCH3 had a poor OS [9], and Li et al. described that patients with high JAG2 expression had a significantly poorer prognosis than those with weak expression [76]. On the other hand, high levels of JAG1 were associated with prolonged OS [74] (Table 6). 

Furthermore, in the present study, the Kaplan–Meier curve of OS and DFS showed that high expression levels of NOTCH3, JAG1, and HEY1 were significantly associated with a worse prognosis (Figure 6 and Figure 7). In contrast, high expression of HES1 was associated with better OS and better DFS. The Kaplan–Meier survival analyses also showed that high expression of DLL4 significantly correlated with a poor OS, but a better DFS. Interestingly, overexpression of NOTCH2 correlated with poor prognosis up to about eight years of follow-up, while after this time point, high expression levels of NOTCH2 were associated with a better prognosis in both OS and DFS. Cox regression analysis revealed that both NOTCH3 and HES1 were independent predictors of OS and DSF in BCa. 

Further analysis of gene combinations suggested that independent of the single gene’s effect on OS/DFS, patients with high expression of combinations involving NOTCH3 were associated with a worse prognosis of OS. This implies that NOTCH3 potentially is the most critical gene for tumorigenesis and progression in BCa.

In addition, our findings are in line with HPA analysis, which also proposed HES1 as a favorable prognostic biomarker with high expression correlates with a better prognosis in urothelial cancer (HPA; https://www.proteinatlas.org/, accessed on 13 December 2020; Table S16) [62]. 

Intriguingly, our OS and DFS analyses revealed an unexpected crossing of the Kaplan–Meier curves of NOTCH2-dependent survival after 8 years and 7.4 years, respectively. No other marker showed such a curve progression (Figure 6 and Figure 7). The interpretation of these unexpected results is difficult. We can think of four scenarios that could explain such a crossing: (i) interaction of the Notch2 receptor with different ligands may lead to different downstream events, of which the causalities are at date not fully understood; (ii) Notch2 receptor activity could be low in those patients, despite high NOTCH2 gene expression; (iii) crosstalk of the Notch 2 pathway with still unrecognized other pathways; or (iv) activation of non-canonical Notch pathways could cause the different results. In any case, much more specific molecular biologic experimental work is required to elucidate those complex events.

In summary, these results support the notion that

(i) Notch pathway-related gene and/or protein expression can serve as diagnostic biomarkers for BCa, with NOTCH4 in particular being the most promising biomarker candidate;
(ii) the Notch pathway may play a critical role in the progression of BCa, while NOTCH3 is the most critical tumorigenesis gene, and HES1 the most protective gene [12,15];
(iii) NOTCH2 and DLL4 have a dual role and function in BCa and the interactions between different receptors, whereas ligands and downstream genes may explain the variable results [72,73];
(iv) all in all, according to the diagnostic, prognostic, and OPLS-DA analyses, NOTCH2, NOTCH3, DLL4, and HES1 are the most essential factors of Notch signaling in BCa, associated with clinical value (Table S17).

### 4.3. Notch Pathway Modulates the Development and Progression of BCa via Immune System

Notch signaling plays a crucial role in T cell development, and potentially plays a dual role in activating or inhibiting T cells [88]. In addition, Notch signaling could induce macrophage phenotype polarization to tumoricidal M1-type macrophage [89], and Notch2 signaling in CD8+ T cells is required for generating potent antitumor cytotoxic T cells [90]. Furthermore, Notch2 function seems to be essential for the proper development of the immune system under normal conditions [91], and gain-of-function mutations play a critical role in tumor immunity [92]. However, inactivation of the Notch2 pathway in CD8+ cytotoxic T cells has also been reported to impair tumor immune response [93]. Numerous studies have explored the role of Notch signaling in anti-tumor immune response in a variety of tumor entities, but thus far none have focused on BCa.

In the present study, GGI network showed that the Notch pathway potentially modulates the immune system in BCa. Additionally, GESA immune signature enrichment analysis also supported the fact that CD4, CD8, and dendritic cell (DC)-associated biological processes are linked to BCa. Using TISIDB, we made a systematic analysis that found the Notch pathway to be significantly associated with the immune system. Our findings confirmed previous studies in the literature, and the results showed that NOTCH2 was broadly and positively associated with the lymphocytes and immunomodulating genes, while NOTCH3 was generally and negatively associated with lymphocytes and immunomodulating genes. On the basis of the coefficient values, we found a moderate positive relationship between the expression level of NOTCH2 with Tcm CD8+ cells, Th2 cells, Mem B cells, Treg cells, and NKT cells. NOTCH2 seems to be the most crucial Notch factor and plays an essential role in modulating the progression of BCa through the immune system.

Moreover, the results also unveiled the Notch pathway modulating the immune system potentially via lymphocytes and immunomodulating genes [63]. Cancer genotypes determine tumor immunophenotypes and tumor escape mechanisms [63]. While gene expression studies cannot uncover molecular mechanisms, our results further strongly support the view that the Notch pathway is involved in regulation of the tumor immunity in BCa.

### 4.4. Notch Pathway Potentially Regulates or Crosstalks with Other Cancer Pathways

The Notch signaling pathway performs crucial roles in maintaining cell fate/proliferation, regulating the stem cell maintenance and differentiation and epithelial–mesenchymal transition (EMT) [94,95,96]. GGI network analysis revealed some exciting functions of Notch factors in BCa, including stem cell development and cell fate determination, indicating peculiar roles for DTX1, DLL3, DLL1, JAG1, and JAG2 in terms of Notch binding, while others are not involved. Furthermore, the canonical pathway and biological process enrichment from GESA also verified that “cell cycle”, “DNA damage telomere stress-induced senescence”, and “mitotic cell cycle checkpoint” are pathways associated with cell proliferation or cell fate and play a critical role in BCa. 

Abdou et al. described the fact that NOTCH1 over-expression in stromal cell potentially activates Oct-4 (one of the crucial regulators of cell self-renewal) and is associated with poor prognosis and liability for recurrence in BCa [96,97,98]. Furthermore, loss-of-function mutations in NOTCH1 and NOTCH2 promote an epithelial–mesenchymal transition (EMT) in BCa, stabilizing the mesenchymal phenotype [7]. However, the question remains as to how the other Notch factors contribute to BCa fate. Our analysis of the relationship between target genes and well-known cancer-related pathways showed that NOTCH4, NOTCH2, NOTCH3, DLL4, DLL1, and JAG1 can either stimulate or inhibit critical cancer-related pathways, e.g., EMT, DNA damage response, cell cycle, apoptosis pathways, TSC/mTOR, RAS/MAPK, and PI3K/AKT (Figure 8F). Thereby, our findings corroborate the notion of the dual role of the Notch pathway depending on a delicate balance between its activating and inhibiting factors.

The tumor suppressor p53 plays an essential role in many malignancies, including promoting progression of BCa [99,100,101,102]. Dueñas et al. found that mutations in the TP53 gene and epigenetically mediated increased p53 protein expression favor the progression and recurrence of non-muscle invasive bladder cancer (NMIBC). Furthermore, mutations in TP53 and expression of splice variants were frequently observed in advanced stages and were associated with a worse prognosis [102]. However, the relationship between the TP53 and the Notch pathway was rarely reported in BCa until now. Consistent with the literature, canonical pathway enrichment based on KEGG and the Reactome Pathway Knowledgebase also showed significant enrichment in “P53 signaling pathway” and “TP53 regulates transcription of cell cycle genes”. Interestingly, our results revealed that NOTCH1, NOTCH2, NOTCH4, DLL1, DLL4, and HES1 expression was significantly different between the patients with TP53 mutation and the cases without.

Taken together, these findings suggest that the Notch pathway plays a crucial role in regulating the progression of BCa through modulation of cell cycle and stemness, directly or indirectly interacting with well-known cancer-related pathways such as p53, TSC/mTOR, RAS/MAPK, and PI3K/AKT. Rather than a single pathway, carcinogenesis is often dependent on multiple pathways [14]. Hence, a better understanding of the related pathways and the molecular crosstalk is pivotal [103]. Consequently, instead of targeting a single pathway, targeting the crosstalk network could be a more meaningful strategy.

### 4.5. Limitations of the Study

Our study allowed us to integrate information of expression levels, mutations, and immune response coupled with pathological and medical information to uncover potential biomarkers and alterations of the Notch pathway in BCa. Our results promote our understanding of the complex influence of the Notch pathway on BCa and may help to improve clinical decision making by proposing certain members of the Notch pathway as potential prognostic biomarkers.

However, our study also has inherent limitations similar to the other bioinformatics approaches due to the restricted availability of cell type-specific data and the still very limited protein expression data. In addition, up until now, functional data as the activation status of proteins/enzymes and ligand–receptor interactions are not recorded in the TCGA and GTEx databases. Bioinformatics try to circumvent this problem by introducing network, gene–gene, and protein–protein interaction analyses on the basis of target gene expression overlaps and predicted interactions. However, specific and extensive experimental in vitro research is needed to confirm the hypothesized functions and to elucidate the complex interactions between the different members of the Notch pathway and the interactions with other cancer-related pathways. Therefore, our study cannot directly answer the urging question of potential therapeutical consequences.

## 5. Conclusions

In conclusion, our bioinformatics analysis provided further evidence for the involvement of the Notch pathway in carcinogenesis, progression, metastasis, and the modulation of the tumor immunity in BCa. The preponderance of the results indicates that NOTCH2, NOTCH3, and DLL4 are potential drivers of Notch signaling in BCa, playing the most crucial role in the progression and development of BCa via modulating the cell cycle and stemness, as well as direct or indirect interactions with well-known cancer-related pathways such as p53, TSC/mTOR, RAS/MAPK, and PI3K/AKT. 

Therefore, the Notch pathway is potentially a candidate target for diagnosis and therapies in BCa. Nevertheless, further work is required to perform the novel strategies, not only concern on the role of single gene/protein but also focusing on function of multi-gene/protein combinations in BCa. Our in silico study is limited to uncover associations and correlations but cannot provide mechanistic insights. Therefore, validation of the cancer biological functions in future in-depth experimental research is needed. Meanwhile, cytological studies are ongoing in our laboratory, seeking to confirm these results. Our findings can help in understanding the function of the core members of the Notch pathway in BCa and can provide further support for the hypothesis of the integral cancer pathway analysis approach.

## Figures and Tables

**Table 1:** Demographics and tumor characteristics of patients with BCa in the present study in terms of TCGA-BLCA.

Patient Characteristics	Classification	Number of Patients	Percent (%)
Age (years), median (IQR)	69 (34–90)	406	100
Gender	Male	299	73.65
	Female	107	26.35
Race	Asian	43	10.59
	Caucasian (White)	323	79.56
	Black African American	23	5.67
	Unknown	17	4.19
Family cancer history	Yes	143	35.22
	No	263	64.78
Histological subtype	Papillary	130	32.02
	Non-papillary	271	66.75
	Unknown	5	1.23
T stage	T0-1	4	0.99
	T2-T2b	118	29.06
	T3-T3b	193	47.54
	T4-T4b	58	14.29
	Tx	1	0.25
	Unknown	32	7.88
Recurrence	Yes	141	34.73
	No	177	43.60
	Unknown	88	21.67
Lymphnode (N) stage	N0	236	58.13
	N1	46	11.33
	N2	75	18.47
	N3	7	1.72
	NX	36	8.87
	Unknown	6	1.48
Metastatic (M) stage	M0	195	48.03
	M1	11	2.71
	MX	197	48.52
	Unknown	3	0.74
Tumor stage	Stage I (T1, N0, M0)	2	0.49
	Stage II (T2a, T2b, N0, M0)	129	31.77
	Stage III (T1-4a, N0-3, M0)	140	34.48
	Stage IV (T1-4b, N1-3, M0-1)	133	32.76
	Unknown	2	0.49
Tumor grade	High grade	383	94.33
	Low grade	20	4.93
	Unknown	3	0.74
Overall status	Dead	179	44.09
	Alive	227	55.91

**Table 2:** Statistically significant difference of expression levels of target genes in subgroups (1).

Target Genes	BCa vs. Control	*p*-Value	Tumor Stage	*p*-Value	Lymph Nodal Metastasis	*p*-Value	Histological Subtypes	*p*-Value	Race	*p*-Value	Gender	*p*-Value
NOTCH1	BCa (↓)	*p* = 6.83 × 10^−7^			C vs. N0 (↓)	*p* = 7.35 × 10^−3^	C vs. PT (↓)	*p* = 1.52 × 10^−3^	CAU vs. AFA (↑)	*p* = 1.04 × 10^−2^		
					C vs. N2 (↓)	*p* = 3.67 × 10^−4^	C vs. NPT (↓)	*p* = 1.46 × 10^−4^				
NOTCH2	BCa (↓)	*p* = 9.46 × 10^−9^	S2 vs. S3 (↑)	*p* = 2.07 × 10^−2^	C vs. N0 (↓)	*p* = 1.00 × 10^−7^	C vs. PT (↓)	*p* = 6.00 × 10^−5^	CAU vs. ASI (↓)	*p* = 2.13 × 10^−4^		
			S2 vs. S4 (↑)	*p* = 2.81 × 10^−2^	C vs. N1 (↓)	*p* = 1.29 × 10^−2^	C vs. NPT (↓)	*p* < 0.000001	AFA vs. ASI (↓)	*p* = 1.00 × 10^−7^		
					C vs. N2 (↓)	*p* = 5.58 × 10^−4^	PT vs. NPT (↑)	*p* = 3.00 × 10^−7^				
NOTCH3	BCa (↑)	*p* = 1.98 × 10^−4^			C vs. N0 (↑)	*p* = 2.20 × 10^−3^	C vs. PT (↑)	*p* = 3.44 × 10^−4^				
					C vs. N1 (↑)	*p* = 6.35 × 10^−4^	C vs. NPT (↑)	*p* = 2.41 × 10^−3^				
					C vs. N2 (↑)	*p* = 1.45 × 10^−2^						
NOTCH4	BCa (↓)	*p* = 5.02 × 10^−12^			C vs. N0 (↓)	*p* < 0.000001	C vs. PT (↓)	*p* = 2.00 × 10^−7^	AFA vs. ASI (↑)	*p* = 2.59 × 10^−3^		
					C vs. N1 (↓)	*p* < 0.000001	C vs. NPT (↓)	*p* < 0.000001				
					C vs. N2 (↓)	*p* = 1.00 × 10^−7^	PT vs. NPT (↓)	*p* = 1.00 × 10^−2^				
					C vs. N3 (↓)	*p* = 7.17 × 10^−4^						
JAG1							PT vs. NPT (↑)	*p* = 6.77 × 10^−4^	CAU vs. AFA (↑)	*p* = 2.93 × 10^−3^		
									AFA vs. ASI (↓)	*p* = 1.47 × 10^−2^		
JAG2			S2 vs. S4 (↓)	*p* = 3.21 × 10^−3^	C vs. N2 (↓)	*p* = 1.06 × 10^−2^			CAU vs. AFA (↑)	*p* = 4.45 × 10^−2^	Male vs. Female (↑)	*p* = 3.20 × 10^−4^
DLL1	BCa (↓)	*p* = 4.67 × 10^−8^			C vs. N0 (↓)	*p* = 1.84 × 10^−4^	C vs. PT (↓)	*p* = 1.00 × 10^−7^				
					C vs. N1 (↓)	*p* = 2.13 × 10^−4^	C vs. NPT (↓)	*p* = 1.85 × 10^−5^				
					C vs. N2 (↓)	*p* = 5.00 × 10^−7^	PT vs. NPT (↑)	*p* = 4.45 × 10^−2^				
					C vs. N3 (↓)	*p* = 1.08 × 10^−2^						
DLL3	BCa (↑)	*p* = 1.02 × 10^−9^			C vs. N0 (↑)	*p* = 3.00 × 10^−7^	C vs. PT (↑)	*p* = 9.51 × 10^−5^				
					C vs. N1 (↑)	*p* = 3.20 × 10^−6^	C vs. NPT (↑)	*p* < 0.000001				
					C vs. N2 (↑)	*p* = 3.80 × 10^−6^	PT vs. NPT (↑)	*p* = 2.26 × 10^−2^			Male vs. Female (↑)	*p* = 3.07 × 10^−2^
					C vs. N3 (↑)	*p* = 2.36 × 10^−3^						
DLL4	BCa (↓)	*p* = 6.36 × 10^−5^			C vs. N0 (↓)	*p* = 7.29 × 10^−3^	C vs. NPT (↓)	*p* = 3.63 × 10^−5^	CAU vs. ASI (↓)	*p* = 1.55 × 10^−02^		
					C vs. N1 (↓)	*p* = 2.19 × 10^−3^	PT vs. NPT (↓)	*p* = 6.46 × 10^−3^	AFA vs. ASI (↓)	*p* = 2.22 × 10^−02^		
HES1	BCa (↑)	*p* = 2.61 × 10^−5^			C vs. N0 (↑)	*p* = 1.00 × 10^−7^	C vs. PT (↑)	*p* < 0.000001	CAU vs. ASI (↓)	*p* = 3.98 × 10^−04^		
					C vs. N1 (↑)	*p* = 3.63 × 10^−3^	C vs. NPT (↑)	*p* = 4.40 × 10^−6^				
					C vs. N2 (↑)	*p* = 2.25 × 10^−5^	PT vs. NPT (↓)	*p* = 5.05 × 10^−3^				

Compared to control group (C; *n* = 40); S2: tumor stage II, *n* = 129; S3: tumor stage III, *n* = 140; S4: tumor stage IV, *n* = 133. Papillary tumors (PT), *n* = 130; non-papillary tumors (NPT), *n* = 271; N0 (no regional lymph node metastasis), *n* = 236; N1 (metastasis in 1 to 3 axillary lymph nodes), *n* = 46; N2 (metastasis in 4 to 9 axillary lymph nodes), *n* = 75; N3 (metastasis in 10 or more axillary lymph nodes), *n* = 7; Caucasian (CAU), *n* = 323; Black or African American (AFA), *n* = 23; Asian (ASI), *n* = 43; male, *n* = 299; female, *n* = 107. “**↑**” means upregulation, “**↓**” means downregulation. The findings with *p*-value < 0.05 were shown above. No significant results were found in HEY1 (data not shown).

**Table 3:** Statistically significant differences of expression levels of target genes in subgroups (2).

Target Genes	Molecular Subtypes				TP53 Mutation Status			
	C-TCGA vs. Subtypes	*p*-Value	BST vs. LT/LIT	*p*-Value	C-TCGA vs. TP53^M^/TP53^WT^	*p*-Value	TP53^M^ vs. TP53^WT^	*p*-Value
NOTCH1	LT (↓)	*p* = 8.20 × 10^−3^	LT (↓)	*p* = 3.31 × 10^−2^	TP53^M^ (↓)	*p* = 6.20 × 10^−3^	TP53^WT^ (↑)	*p* = 3.91 × 10^−7^
			LT (↓)	*p* = 6.16 × 10^−4^				
NOTCH2	LT (↓)	*p* = 1.50 × 10^−3^	LT (↓)	*p* = 1.14 × 10^−3^	TP53^M^ (↓)	*p* = 3.46 × 10^−2^	TP53^WT^ (↓)	*p* = 4.34 × 10^−2^
	LIT (↓)	*p* = 3.68 × 10^−2^			TP53^WT^ (↓)	*p* = 1.17 × 10^−2^		
	LPT (↓)	*p* = 5.44 × 10^−3^						
NOTCH3	BST (↑)	*p* = 5.69 × 10^−5^	LIT (↑)	*p* = 4.05 × 10^−2^	TP53^M^ (↑)	*p* = 5.83 × 10^−4^		
	LT (↑)	*p* = 1.26 × 10^−3^			TP53^WT^ (↑)	*p* = 2.22 × 10^−5^		
	LIT (↑)	*p* = 1.06 × 10^−2^						
	LPT (↑)	*p* = 8.22 × 10^−5^						
NOTCH4	BST (↓)	*p* = 1.47 × 10^−2^	LIT (↑)	*p* = 9.78 × 10^−3^	TP53^M^ (↓)	*p* = 3.02 × 10^−2^	TP53^WT^ (↑)	*p* = 4.62 × 10^−3^
JAG1	BST (↑)	*p* = 1.10 × 10^−6^	LT (↓)	*p* = 9.77 × 10^−5^	TP53^M^ (↑)	*p* = 3.95 × 10^−2^		
			LIT (↓)	*p* = 1.13 × 10^−7^				
JAG2	NET (↑)	*p* = 2.09 × 10^−2^	LT (↓)	*p* = 5.25 × 10^−12^	TP53^M^ (↑)	*p* = 1.32 × 10^−3^		
	BST (↑)	*p* = 4.76 × 10^−8^	LIT (↓)	*p* = 5.87 × 10^−9^	TP53^WT^ (↑)	*p* = 3.98 × 10^−4^		
	LPT (↑)	*p* = 3.99 × 10^−2^						
DLL1	LT (↓)	*p* = 3.06 × 10^−3^	LT (↓)	*p* = 1.44 × 10^−4^			TP53^WT^ (↓)	*p* = 2.86 × 10^−2^
	LIT (↓)	*p* = 5.54 × 10^−3^	LIT (↓)	*p* = 7.74 × 10^−5^				
	LPT (↓)	*p* = 1.31 × 10^−3^						
DLL3	BST (↑)	*p* = 3.48 × 10^−7^	LIT (↓)	*p* = 1.96 × 10^−3^	TP53^M^ (↑)	*p* = 1.00 × 10^−2^		
	LT (↑)	*p* = 4.68 × 10^−2^			TP53^WT^ (↑)	*p* = 1.75 × 10^−6^		
	LIT (↑)	*p* = 9.80 × 10^−3^						
	LPT (↑)	*p* = 5.16 × 10^−3^						
DLL4			LT (↑)	*p* = 1.77 × 10^−2^			TP53^WT^ (↑)	*p* = 4.02 × 10^−3^
			LIT (↑)	*p* = 4.46 × 10^−3^				
HES1	LT (↑)	*p* = 3.51 × 10^−3^	LT (↑)	*p* = 3.97 × 10^−5^	TP53^WT^ (↑)	*p* = 2.30 × 10^−2^	TP53^WT^ (↑)	*p* = 2.11 × 10^−2^
	LIT (↑)	*p* = 4.79 × 10^−2^	LIT (↑)	*p* = 2.18 × 10^−3^				
	LPT (↑)	*p* = 3.14 × 10^−6^						
HEY1	NET (↑)	*p* = 4.46 × 10^−2^	LT (↑)	*p* = 2.20 × 10^−2^	TP53^M^ (↑)	*p* = 1.16 × 10^−3^		
	BST (↑)	*p* = 5.74 × 10^−3^			TP53^WT^ (↑)	*p* = 1.63 × 10^−6^		
	LT (↑)	*p* = 1.68 × 10^−3^						
	LIT (↑)	*p* = 1.50 × 10^−2^						

Non-cancerous tissue from TCGA-BLCA (C-TCGA), *n* = 19; neuronal tumors (NET), *n* = 20; basal squamous tumors (BST), *n* = 142; luminal tumors (LT), *n* = 26; luminal-infiltrated tumors (LIT), *n* = 78; luminal-papillary tumors (LPT), *n* = 142; TP53^M^ (TP53-mutant), *n* = 193; TP53^WT^ (TP53-wild type), *n* = 215; upregulation (**↑**)**;** downregulation (**↓**); *p*-value < 0.05; UALCAN (http://ualcan.path.uab.edu, accessed on 20 December 2020) [44].

**Table 4:** Risk assessment of Notch components on OS.

	Univariate Test				Multivariate Test		
Gene	HR	CI95	*p*-Value	Gene	HR	CI95	*p*-Value
NOTCH1	1.21	0.9–1.63	0.208				
NOTCH2	1.44	1.06–1.96	0.018 *	NOTCH2	1.27	0.92–1.76	0.151
NOTCH3	1.6	1.2–2.15	0.002 **	NOTCH3	1.65	1.2–2.25	0.002 **
NOTCH4	1.35	0.85–2.15	0.204				
JAG1	4.26	1.98–9.18	0	JAG1	3.69	1.67–8.15	0.001 **
JAG2	1.25	0.88–1.77	0.221				
DLL1	1.47	0.87–2.5	0.154				
DLL3	0.89	0.64–1.24	0.483				
DLL4	1.53	1.11–2.1	0.009 **	DLL4	1.54	1.11–2.14	0.009 **
HES1	0.57	0.41–0.8	0.001 **	HES1	0.56	0.39–0.81	0.002 **
HEY1	2.26	1–5.11	0.045 *	HEY1	1.63	0.7–3.77	0.255

Cox regression analysis; hazard ratio = HR; 95% confidence interval = CI95%; * = *p*-value < 0.05; ** = *p*-value < 0.01.

**Table 5:** Risk assessment of Notch components on DFS.

	Univariate Test				Multivariate Test		
Gene	HR	CI95	*p*-Value	Gene	HR	CI95	*p*-Value
NOTCH1	1.18	0.85–1.65	0.322				
NOTCH2	1.53	1.09–2.16	0.014 *	NOTCH2	1.3	0.91–1.85	0.152
NOTCH3	1.46	1.05–2.04	0.026 *	NOTCH3	1.52	1.08–2.15	0.016 *
NOTCH4	0.88	0.63–1.23	0.447				
JAG1	1.9	1.02–3.52	0.042 *	JAG1	1.42	0.75–2.68	0.28
JAG2	1.66	1.07–2.58	0.023 *	JAG2	1.63	1.05–2.55	0.03 *
DLL1	1.12	0.81–1.56	0.494				
DLL3	1.19	0.85–1.67	0.3				
DLL4	0.65	0.44–0.95	0.028 *	DLL4	0.68	0.46–1.01	0.056
HES1	0.56	0.39–0.82	0.003 **	HES1	0.59	0.4–0.88	0.01 *
HEY1	1.97	1–3.87	0.046 *	HEY1	1.67	0.84–3.34	0.146

Cox regression analysis; hazard ratio = HR; 95% confidence interval = CI95%; * = *p*-value < 0.05; ** = *p*-value < 0.01.

**Table 6:** The dual role of Notch receptors and ligands in BCa.

Name	Role in BCa	Description
NOTCH1	Suppression *(↓)	Activated NOTCH1 suppressed BCa in vitro and in vivo by reducing ERK1 and ERK2(ERK1/2) [8]. Suppression of NOTCH1 may be advantageous for tumor progression [6].
	Oncogene (↑)	NOTCH1 knockdown led to cancer cell growth and significantly inhibited growth and proliferation [72].
NOTCH2	Suppression *(↓)	NOTCH2 was significantly downregulated in BCa [6,8]. Inactivation of NOTCH2 favors the process of EMT and promotes BCa progression [7].
	Oncogene (↑)	High rate of NOTCH2 copy number gain and over-expression in BCa, and the activation of NOTCH2 promotes metastasis, resulting in poor survival [5].
NOTCH3	Suppression (↓)	Deletion of the intracellular domain of NOTCH3 leads to negative function in BCa, i.e., turning it into potential tumor-suppressive gene [8].
	Oncogene*(↑)	Significantly upregulated NOTCH3 enhanced the BCa growth and chemoresistance in urothelial carcinoma with worse survival [9].
NOTCH4	Suppression * (↓)	NOTCH4 was significantly downregulated in BCa, especially in the T1 stage of bladder tumor. However, in patients diagnosed with muscle-invasive bladder tumor (MIBC), high NOTCH4 expression correlated with poor survival and more vascular invasion [73].
	Oncogene	NA
JAG1	Suppression (↓)	JAG1 significantly decreased in BCa and was associated with poor prognosis [74].
	Oncogene (↑)	miR-489 directly suppressed JAG1 expression, inhibiting the proliferation and invasion of human bladder cancer cells [75].
JAG2	Suppression	NA
	Oncogene (↑)	JAG2 was overexpressed in BCa and was significantly associated with the metastasis and recurrence [76].
DLL1	Suppression *(↓)	DLL1 was significantly decreased in BCa. Supposed to be a suppressive gene [6].
	Oncogene	NA
DLL3	Suppression	NA
	Oncogene * (↑)	DLL3 was significantly upregulated in small cell components correlated with worse clinical outcomes in small cell bladder cancer (SCBC) [77].
DLL4	Suppression	NA
	Oncogene (↑)	DLL4 was significantly upregulated in BCa, and the expression of DLL4 was found to be associated with vascular differentiation in BCa [78].

(*) Expression level consistent with the present study. NA means no literature retrieved. The expression level alteration in representative studies: upregulation in BCa (**↑**); downregulation in BCa (**↓**).

## Data Availability

The data presented in this study are openly available in the Cancer Genome Atlas Program (TCGA), the Genotype-Tissue Expression (GTEx), and the Human Protein Atlas (HPA). Furthermore, the other web servers provide easy access to publicly available cancer OMICS data (TCGA, GTEx, et al.) and allow users to identify biomarkers or to perform in silico validation of potential genes, including GEPIA, GeneMANIA, Oncomine, GSCALite, UALCAN, and TISIDB for analyzing RNA sequencing expression and other data projects via a standard processing pipeline, and following the guidelines in the Data Use Certification Agreement.

## Supplementary Materials

The following are available online at https://www.mdpi.com/article/10.3390/cancers13123089/s1: File S1: List of full gene names and description of “GSE20727 CTRL vs. ROS INH and DNFB allergen-treated DC DN”. Figure S1: Data before and after normalization. Figure S2: Expression of Notch-related genes in relation to tumor TNM stage. Figure S3: Lymph node status. Figure S4: Histologic type (papillary vs. non-papillary). Figure S5: Molecular subtypes. Figure S6: ROC analysis of JAG1, JAG2, and HEY1. Figure S7: Landmark analysis of Notch2 in OS and DFS. Figure S8: Analysis of combination in OS and DFS analysis. Figure S9: Body map construction and Oncomine meta-analysis and IHC of Notch pathway. Figure S10: The expression level of target genes in normal bladder tissue. Table S1: Demographics of controls in present study based on GTEx and TCGA-BLCA. Table S2: Diagnostic values of the components of Notch pathway. Table S3: Correlation between the components of Notch pathway and lymphocytes. Table S4: Correlation between the components of Notch pathway and immunoinhibitors. Table S5: Correlation between the components of Notch pathway and immunostimulators. Table S6: OS/DFS analysis of target genes and combinations in BCa. Table S7: Gene network analysis on interactions. Table S8: Gene network analysis results of function. Table S9: GSEA-canonical pathway enrichment based on KEGG. Table S10: GSEA canonical pathway analysis based on Reactome. Table S11: GSEA biological process of GO analysis. Table S12: GSEA immunologic signature gene set enrichment. Table S13: Meta-analysis of Notch BCa from Oncomine database. Table S14: Venn diagram data of subgroup analysis based on Notch receptors. Table S15: Venn diagram data of subgroup analysis based on Notch ligands and downstream genes. Table S16: Overall survival analysis of target genes in BCa based on HPA. Table S17: Venn diagram data of diagnostic, prognostic, and OPLS-DA analysis.

## Abbreviations

AFA	Black or African American
ASI	Asian
AUC	Accuracy
BCa	Bladder cancer
BP	Biological process
BST	Basal squamous tumors
CAU	Caucasian
DC	Dendritic cell
DFS	Disease-free survival
DLL	Delta-like canonical Notch ligand
EMT	Epithelial to mesenchymal transition
ER	Hormone estrogen receptor
FC	Fold change
FDR	False discovery rate
FWER	Familywise error rate
GEPIA	Gene expression profiling interactive analysis
GGI	Gene‒gene interaction
GO	Gene ontology
GSEA	Gene set enrichment analysis
GTEx	Gene expression omnibus
HPA	Human protein atlas
HR	Hormone androgen receptor
IHC	Immunochemistry
JAG	Serrate-like jagged canonical Notch ligand
KEGG	Kyoto Encyclopedia of Genes and Genomes
LIT	Luminal-infiltrated tumors
LPT	Luminal-papillary tumors
LT	Luminal tumors
MIBC	Muscle-invasive bladder cancer
NCBI	National Center for Biotechnology Information
NES	Normalized enrichment score
NET	Neuronal tumors
NMIBC	Non-muscle-invasive bladder cancer
NOTCH	Neurogenic locus Notch homologue
NPT	Non-papillary tumor
OS	Overall survival
OPLA-DA	Orthogonal partial least squares discriminant analysis
PT	Papillary tumor
RTK	Receptor tyrosine kinase signaling
RAS/MAPK	Ras/mitogen-activated protein kinase pathway
PCA	Principal component analysis
ROC	Receiver operating characteristic
SCBC	Small cell bladder cancer
TCGA-BLCA	Cancer genome atlas bladder cancer
TSC-mTOR	Tuberous sclerosis complex–mammalian target of rapamycin (TSC-mTOR) pathway
TISIDB	Tumor and immune system interaction database
TPM	Transcripts per million
