# Supplementary material for: A Comprehensive Bioinformatics Analysis of Notch Pathways in Bladder Cancer
Source: Cancers (Basel). 2021 Jun 21;13(12):3089. doi: 10.3390/cancers13123089 (PMC8235546; doi:10.3390/cancers13123089)
Supplement: Supplementary file 1 [file cancers-13-03089-s001.zip › cancers-1227629-supplementary/Supplementary Files Proofs/edJNCancers-1227629-supplementary.pdf]

cancers

MDPI

Article

Supplementary Material: A Comprehensive Bioinformatics Analysis of Notch Pathways in Bladder Cancer

Chuan Zhang [1,2], Mandy Berndt-Paetz [1] and Jochen Neuhaus [1,*]

File S1. list of full gene names and description of "GSE20727 CTRL vs ROS INH and DNFB allergen treated DC DN".

Gene	Full name of protein
APH1A	Gamma-secretase subunit APH-1A
APH1B	Gamma-secretase subunit APH-1B
DLK1	Protein delta homolog 1
DLL1	Delta-like protein 1
DLL3	Delta-like protein 3
DLL4	Delta-like protein 4
DNER	Delta and Notch-like epidermal growth factor-related receptor
DTX1	E3 ubiquitin-protein ligase DTX1
GNPTAB	N-acetylglucosamine-1-phosphotransferase subunits alpha/beta
HEG1	Protein HEG homolog 1
HES1	Hairy and enhancer of split 1
HES6	Hairy and enhancer of split 6
HEY1	Hairy/enhancer-of-split related with YRPW motif protein 1
JAG1	Protein jagged-1
JAG2	Protein jagged-2
LFNG	Beta-1,3-N-acetylglucosaminyltransferase lunatic fringe
LTBP2	Latent-transforming growth factor beta-binding protein 2
MAML1	Mastermind-like protein 1
MAML2	Mastermind-like protein 2
MAML3	Mastermind-like protein 3
MFNG	Beta-1,3-N-acetylglucosaminyltransferase manic fringe
NCSTN	Nicastrin
NEURL1	E3 ubiquitin-protein ligase NEURL1
NEURL1B	E3 ubiquitin-protein ligase NEURL1B
NOTCH1	Neurogenic locus notch homolog protein 1
NOTCH2	Neurogenic locus notch homolog protein 2
NOTCH3	Neurogenic locus notch homolog protein 3
NOTCH4	Neurogenic locus notch homolog protein 4
POFUT1	GDP-fucose protein O-fucosyltransferase 1
RP11-458D21.5	Uncharacterized protein
SNED1	Sushi, nidogen and EGF-like domain-containing protein 1

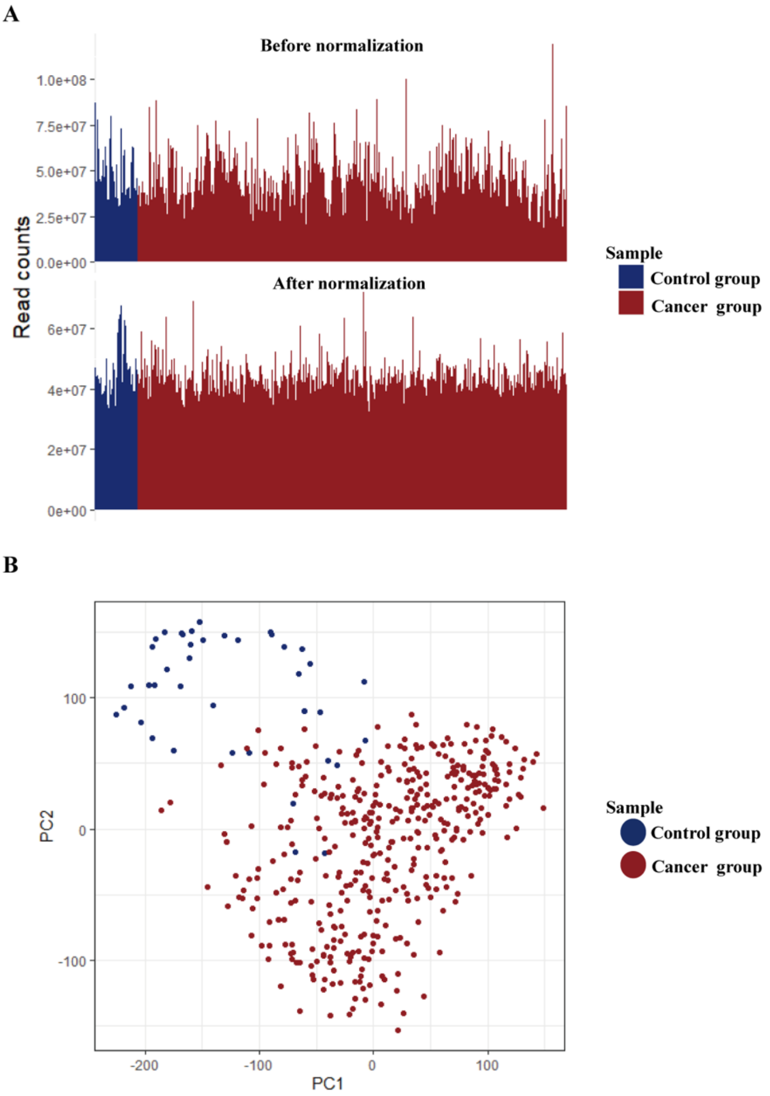

Figure S1. Distribution of data before and after normalization (A). and principle-component analysis of the data after the normalization.

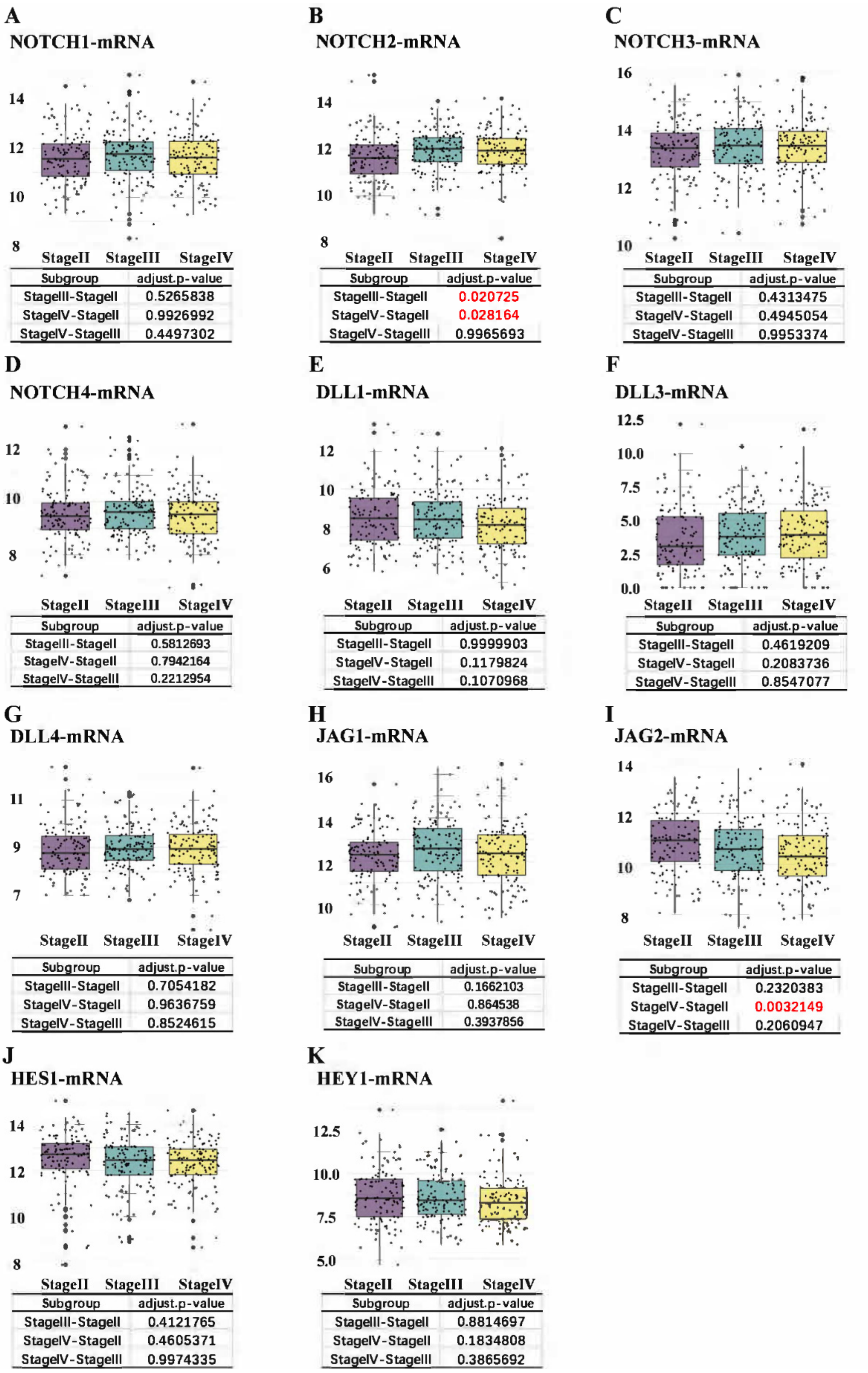

Figure S2. Expression of Notch-related genes in relation to tumor TNM-stage; box indicates 25%-75% percentile, line = median, whiskers indicate 95% CI; significant *p*-values ($p < 0.05$) are colored red.

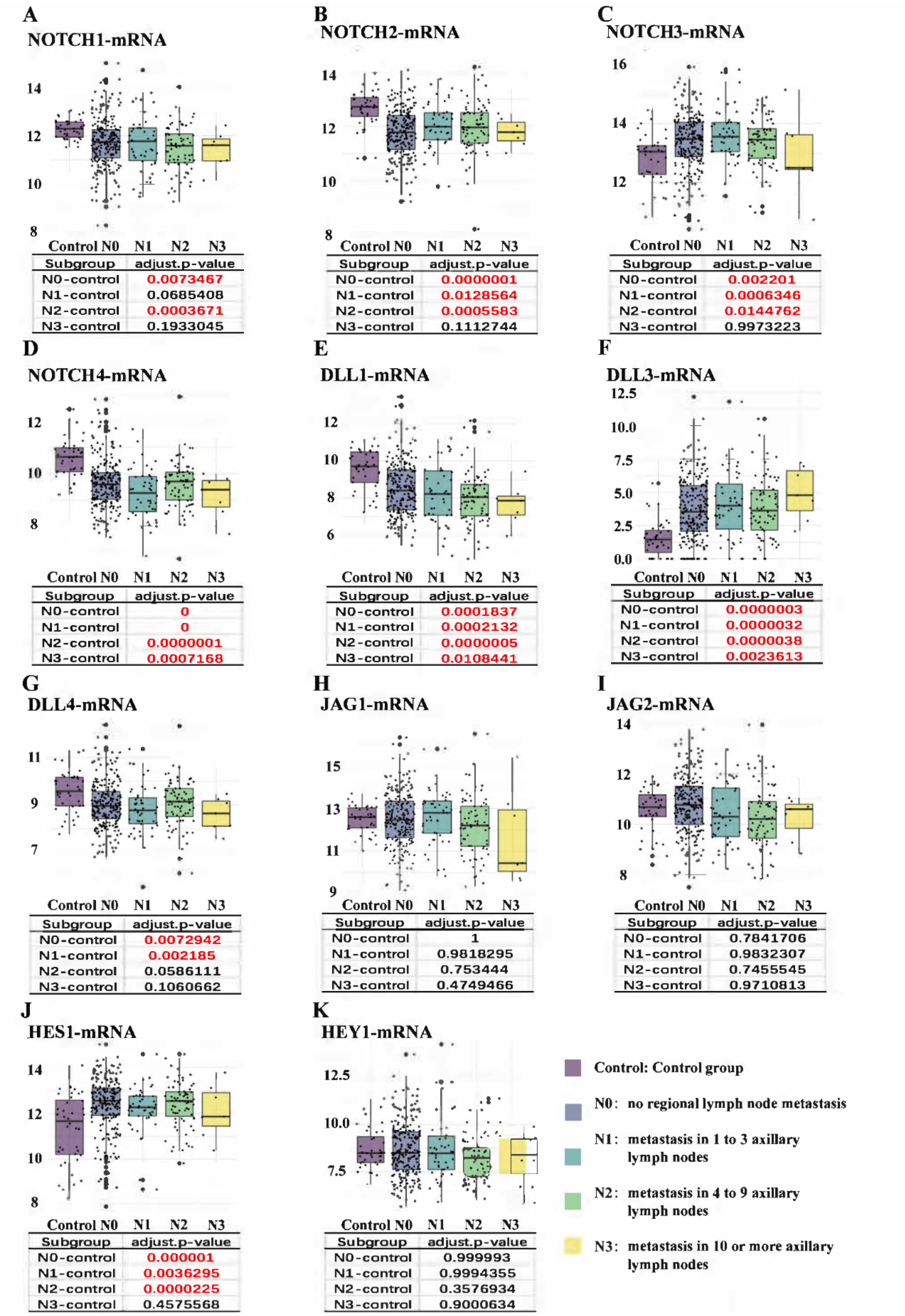

Figure S3. Expression of Notch-related genes in relation to lymph node status; box indicates 25%-75% percentile, line = median, whiskers indicate 95% CI; significant *p*-values ($p < 0.05$) are colored red.

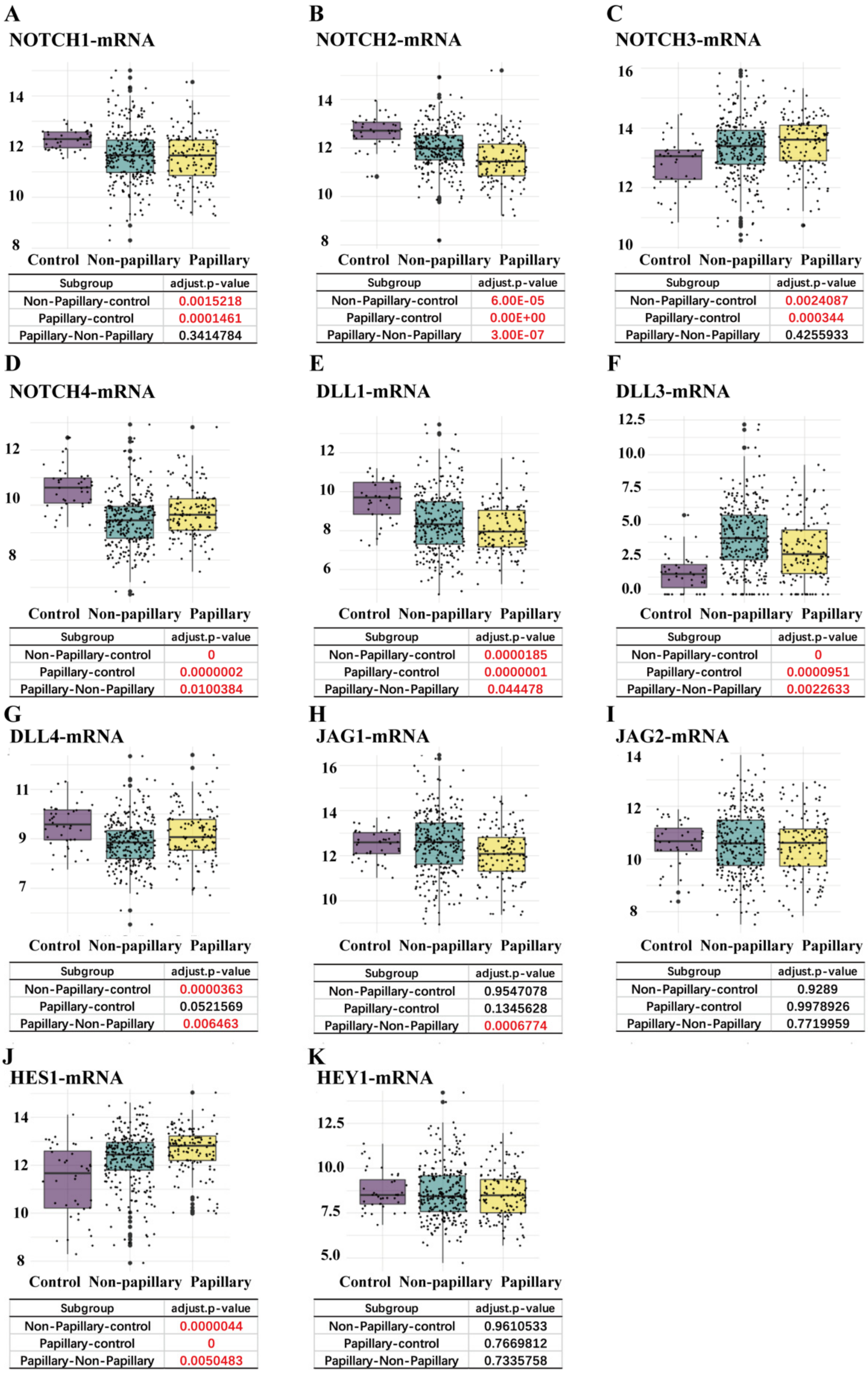

A NOTCH1-mRNA

Subgroup	adjust.p-value
Non-Papillary-control	0.0015218
Papillary-control	0.0001461
Papillary-Non-Papillary	0.3414784

B NOTCH2-mRNA

Subgroup	adjust.p-value
Non-Papillary-control	6.00E-05
Papillary-control	0.00E+00
Papillary-Non-Papillary	3.00E-07

C NOTCH3-mRNA

Subgroup	adjust.p-value
Non-Papillary-control	0.0024087
Papillary-control	0.000344
Papillary-Non-Papillary	0.4255933

D NOTCH4-mRNA

Subgroup	adjust.p-value
Non-Papillary-control	0
Papillary-control	0.0000002
Papillary-Non-Papillary	0.0100384

E DLL1-mRNA

Subgroup	adjust.p-value
Non-Papillary-control	0.0000185
Papillary-control	0.0000001
Papillary-Non-Papillary	0.044478

F DLL3-mRNA

Subgroup	adjust.p-value
Non-Papillary-control	0
Papillary-control	0.0000951
Papillary-Non-Papillary	0.0022633

G DLL4-mRNA

Subgroup	adjust.p-value
Non-Papillary-control	0.0000363
Papillary-control	0.0521569
Papillary-Non-Papillary	0.006463

H JAG1-mRNA

Subgroup	adjust.p-value
Non-Papillary-control	0.9547078
Papillary-control	0.1345628
Papillary-Non-Papillary	0.0006774

I JAG2-mRNA

Subgroup	adjust.p-value
Non-Papillary-control	0.9289
Papillary-control	0.9978926
Papillary-Non-Papillary	0.7719959

J HES1-mRNA

Subgroup	adjust.p-value
Non-Papillary-control	0.0000044
Papillary-control	0
Papillary-Non-Papillary	0.0050483

K HEY1-mRNA

Subgroup	adjust.p-value
Non-Papillary-control	0.9610533
Papillary-control	0.7669812
Papillary-Non-Papillary	0.7335758

Figure S4. Expression of Notch-related genes in relation to histologic type (papillary vs. non-papillary); box indicates 25%-75% percentile, line = median, whiskers indicate 95% CI; significant *p*-values ($p < 0.05$) are colored red.

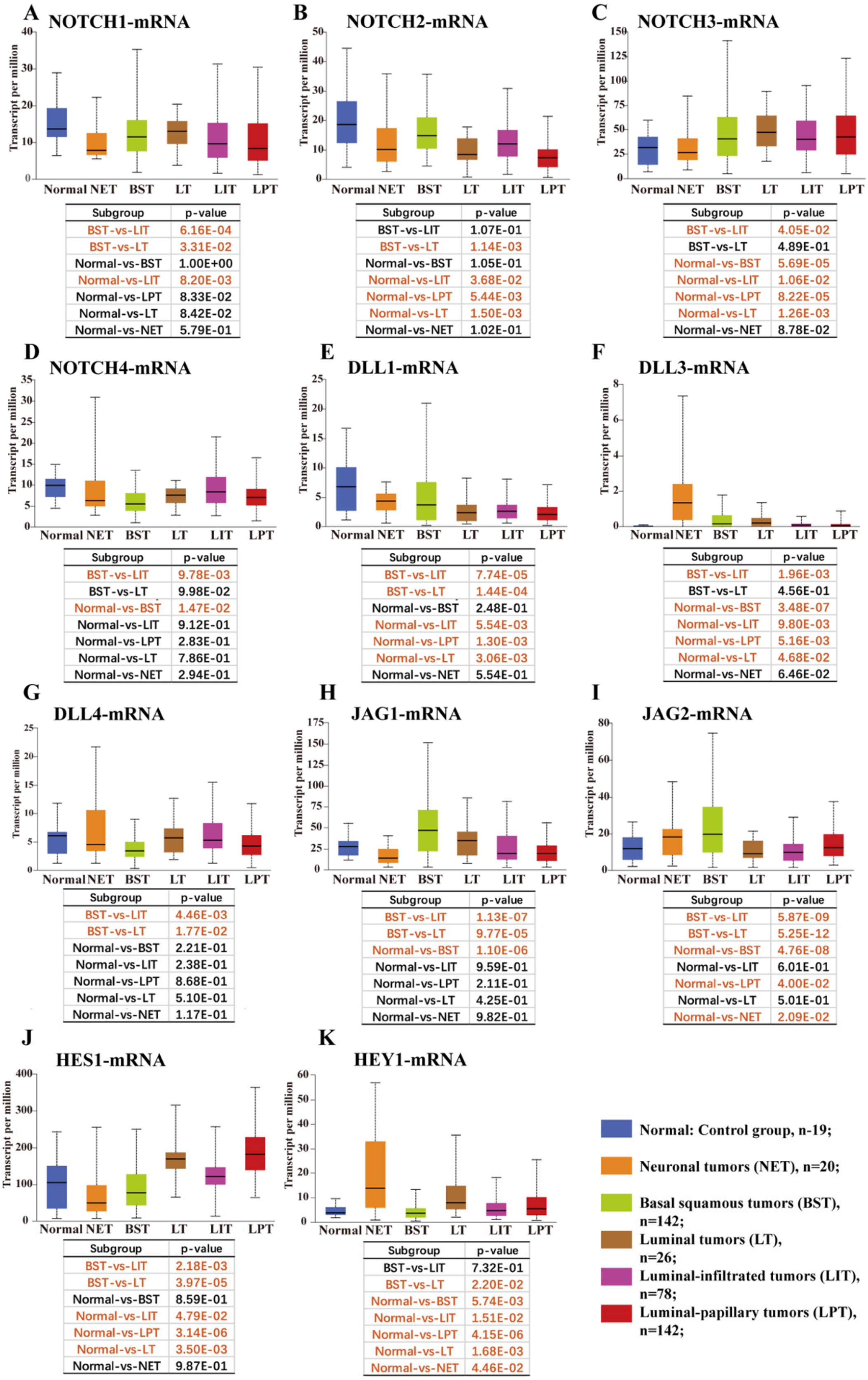

A

Subgroup	p-value
BST-vs-LIT	6.16E-04
BST-vs-LT	3.31E-02
Normal-vs-BST	1.00E+00
Normal-vs-LIT	8.20E-03
Normal-vs-LPT	8.33E-02
Normal-vs-LT	8.42E-02
Normal-vs-NET	5.79E-01

B

Subgroup	p-value
BST-vs-LIT	1.07E-01
BST-vs-LT	1.14E-03
Normal-vs-BST	1.05E-01
Normal-vs-LIT	3.68E-02
Normal-vs-LPT	5.44E-03
Normal-vs-LT	1.50E-03
Normal-vs-NET	1.02E-01

C

Subgroup	p-value
BST-vs-LIT	4.05E-02
BST-vs-LT	4.89E-01
Normal-vs-BST	5.69E-05
Normal-vs-LIT	1.06E-02
Normal-vs-LPT	8.22E-05
Normal-vs-LT	1.26E-03
Normal-vs-NET	8.78E-02

D

Subgroup	p-value
BST-vs-LIT	9.78E-03
BST-vs-LT	9.98E-02
Normal-vs-BST	1.47E-02
Normal-vs-LIT	9.12E-01
Normal-vs-LPT	2.83E-01
Normal-vs-LT	7.86E-01
Normal-vs-NET	2.94E-01

E

Subgroup	p-value
BST-vs-LIT	7.74E-05
BST-vs-LT	1.44E-04
Normal-vs-BST	2.48E-01
Normal-vs-LIT	5.54E-03
Normal-vs-LPT	1.30E-03
Normal-vs-LT	3.06E-03
Normal-vs-NET	5.54E-01

F

Subgroup	p-value
BST-vs-LIT	1.96E-03
BST-vs-LT	4.56E-01
Normal-vs-BST	3.48E-07
Normal-vs-LIT	9.80E-03
Normal-vs-LPT	5.16E-03
Normal-vs-LT	4.68E-02
Normal-vs-NET	6.46E-02

G

Subgroup	p-value
BST-vs-LIT	4.46E-03
BST-vs-LT	1.77E-02
Normal-vs-BST	2.21E-01
Normal-vs-LIT	2.38E-01
Normal-vs-LPT	8.68E-01
Normal-vs-LT	5.10E-01
Normal-vs-NET	1.17E-01

H

Subgroup	p-value
BST-vs-LIT	1.13E-07
BST-vs-LT	9.77E-05
Normal-vs-BST	1.10E-06
Normal-vs-LIT	9.59E-01
Normal-vs-LPT	2.11E-01
Normal-vs-LT	4.25E-01
Normal-vs-NET	9.82E-01

I

Subgroup	p-value
BST-vs-LIT	5.87E-09
BST-vs-LT	5.25E-12
Normal-vs-BST	4.76E-08
Normal-vs-LIT	6.01E-01
Normal-vs-LPT	4.00E-02
Normal-vs-LT	5.01E-01
Normal-vs-NET	2.09E-02

J

Subgroup	p-value
BST-vs-LIT	2.18E-03
BST-vs-LT	3.97E-05
Normal-vs-BST	8.59E-01
Normal-vs-LIT	4.79E-02
Normal-vs-LPT	3.14E-06
Normal-vs-LT	3.50E-03
Normal-vs-NET	9.87E-01

K

Subgroup	p-value
BST-vs-LIT	7.32E-01
BST-vs-LT	2.20E-02
Normal-vs-BST	5.74E-03
Normal-vs-LIT	1.51E-02
Normal-vs-LPT	4.15E-06
Normal-vs-LT	1.68E-03
Normal-vs-NET	4.46E-02

Figure S5. Expression of Notch-related genes in relation to molecular subtypes; box indicates 25%-75% percentile, line = median, whiskers indicate 95% CI; significant p-values ($p < 0.05$) are colored red.

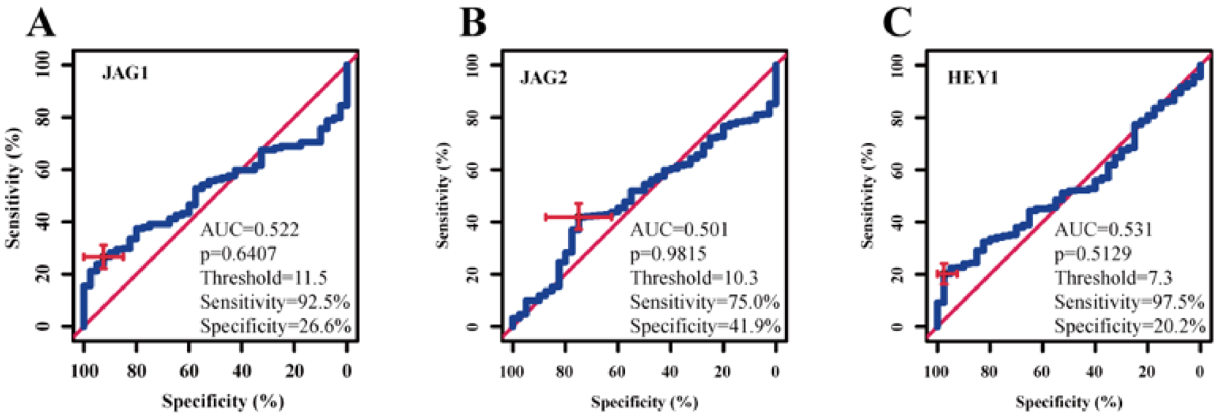

Figure S6. Diagnostic value of Notch pathway related genes. (A) JAG1, (B) JAG2, (C) HEY1.

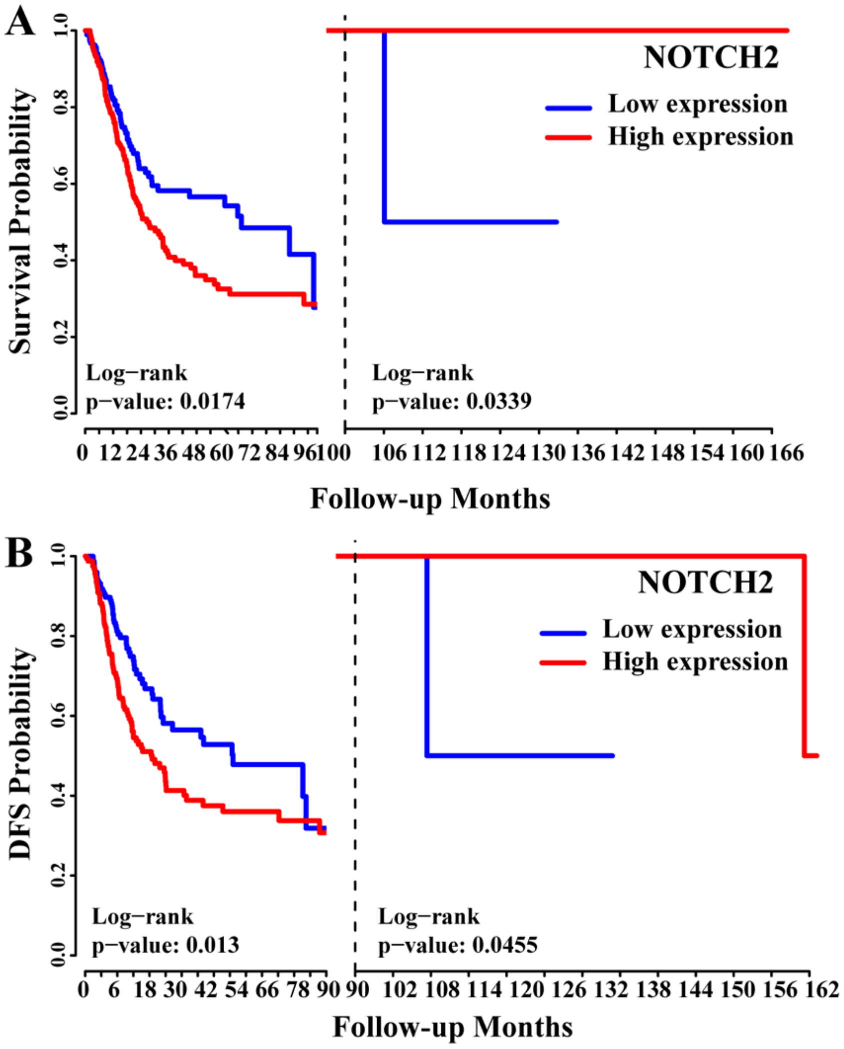

Figure S7. Landmark Analysis and Time Dependent Covariates of NOTCH2. Data from TCGA-BLCA, *n* = 406; dead, *n*= 179, alive, *n* = 227; Recurrence, *n*= 141, not recurrence, *n* = 177; see Table S6 for threshold (cut-off value) used to define the high and low levels of risk; Y-axis = Overall Survival probability, X-axis = follow up in months, high expression (red line), low expression (blue line).

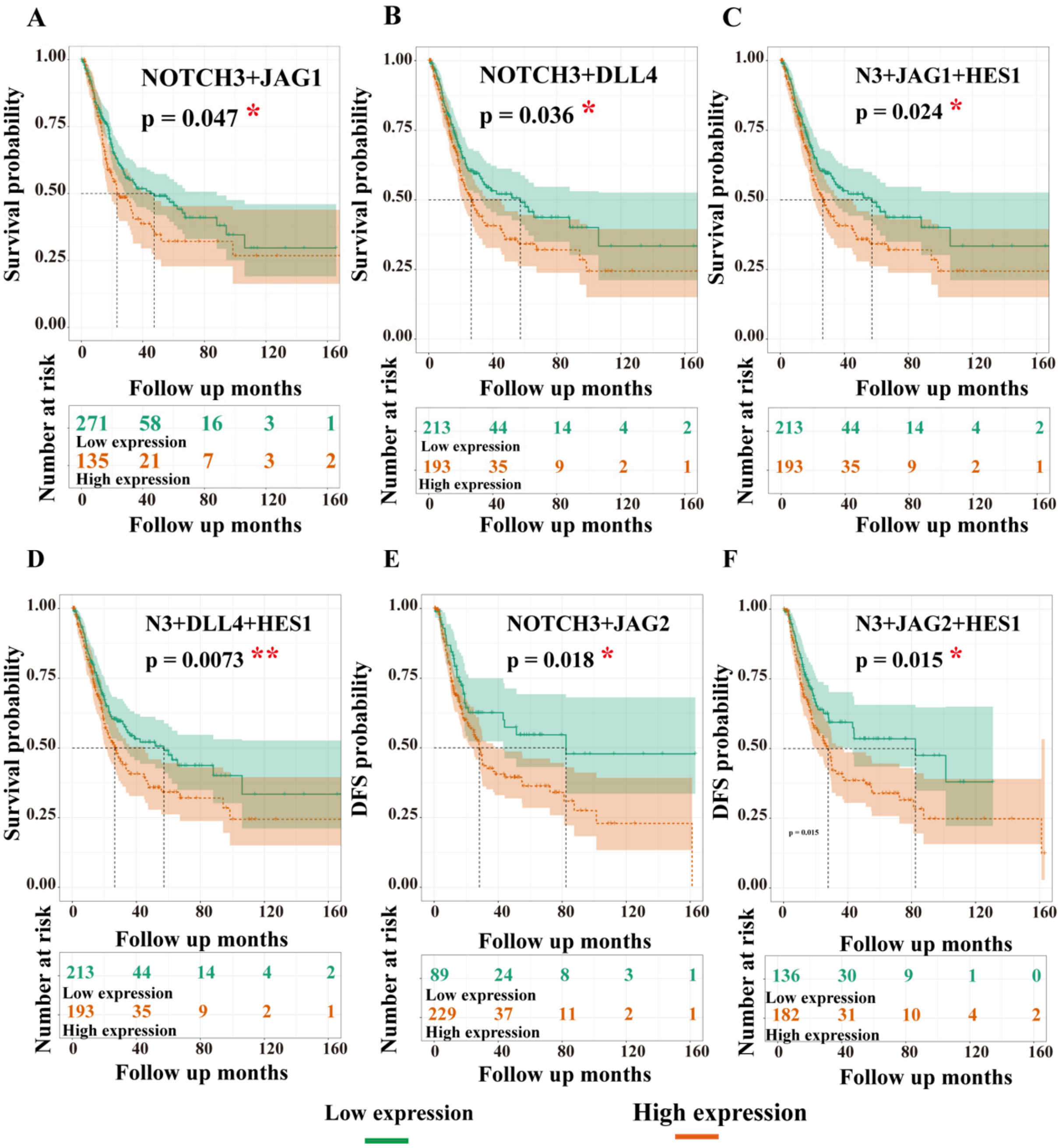

Figure S8. OS/DFS analyses of the combination of the target genes. data from TCGA-BLCA, *n* = 406; dead, *n*= 179, alive, *n* = 227; Recurrence, *n* = 141, not recurrence, *n* = 177; see Table S6 for threshold (cut-off value) used to define the high and low levels of risk; Y-axis = Overall Survival probability, X-axis = follow up in months, high expression (orange line), low expression (green line); the number at risk in high- and low-expression group are listed in the tables below the survival curve; significance levels are marked as: *$p < 0.05$, **$p < 0.01$, ***$p < 0.001$, ****$p < 0.0001$.

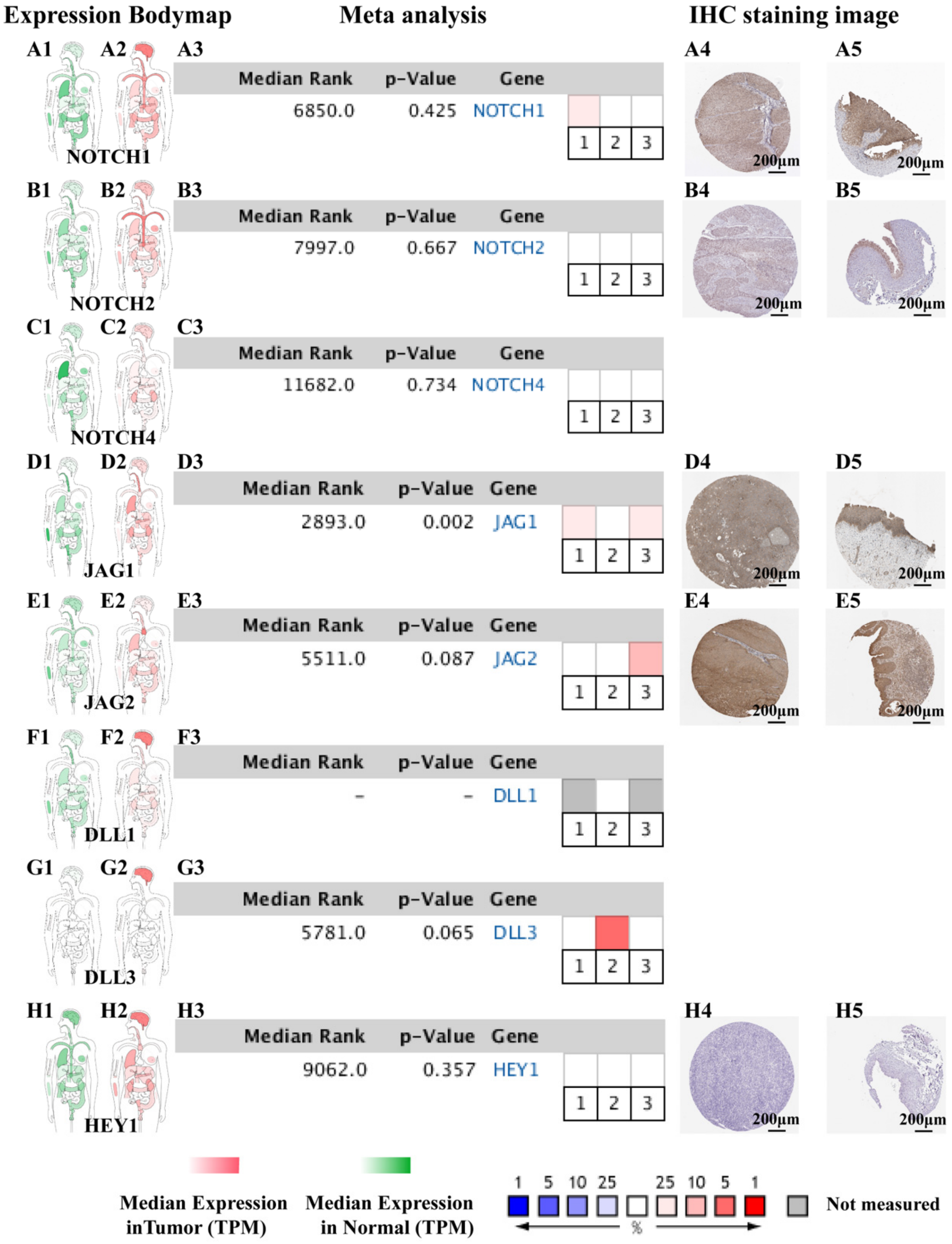

Figure S9. Oncomine meta-analysis of representative genes in BCa vs non-cancerous tissue, and the body maps depicting the mRNA expression levels of the target genes. (i) Oncomine meta-analysis of NOTCH1 (A), NOTCH2 (B), NOTCH4 (C), JAG1 (D), JAG2(E), DLL1 (F), DLL3 (G), and HEY1 (H) in BCa vs non-cancerous bladder tissue (https://www.oncomine.org/, accessed on Dec.13. 2020) [65]; heat maps of NOTCH3, DLL4 and HES1 demonstrating the gene expression in BCa samples vs. non-cancerous tissues as reported in (1) Dyrskjot et al., Cancer Res, 2004 [71], infiltrating bladder

urothelial carcinoma (*n* = 13) vs. normal (*n* = 14); (2) Lee et al., J Clin Oncol, 2010 [73], infiltrating bladder urothelial carcinoma (*n* = 62) vs. normal (*n* = 10); (3) Sanchez-Carbayo et al., J Clin Oncol, 2006 [72], infiltrating bladder urothelial carcinoma (*n* = 72) vs. normal (*n* = 52); right hand: median rank (median rank of the gene across each of the analyses); *p*-value (*p*-value for the median-ranked analysis); color of the boxes indicates the percentile of the z-transformed expression level of the gene in the particular study; left hand: *p* = (*p*-value reported in each of the studies); NA and the box was filled in grey mean not measured in the study. (ii) The median expression of NOTCH1 (A1, A2), NOTCH2 (B1,B2), NOTCH4 (C1,C2), JAG1 (D1,D2), JAG2(E1,E2), DLL1 (F1,F2), DLL3 (G1,G2), and HEY1 (H1,H2), in normal tissues (green) and tumours (red) in various organs; body maps constructed based on the GEPIA database (http://gepia.cancer-pku.cn, accessed on Nov. 12. 2020) [61], transcripts per million (TPM); (iii) IHC images credit: Human Protein Atlas (https://www.proteinatlas.org/, accessed on Dec.13. 2020) [65]; IHC staining of NOTCH1 (A4) NOTCH2 (B4), JAG1(D4), JAG2 (E4) in cancerous; NOTCH1 (A5), NOTCH2 (B5), JAG1 (D5), JAG2 (E5) in normal tissue(brown precipitates); HEY1 were negative both in cancer and normal.

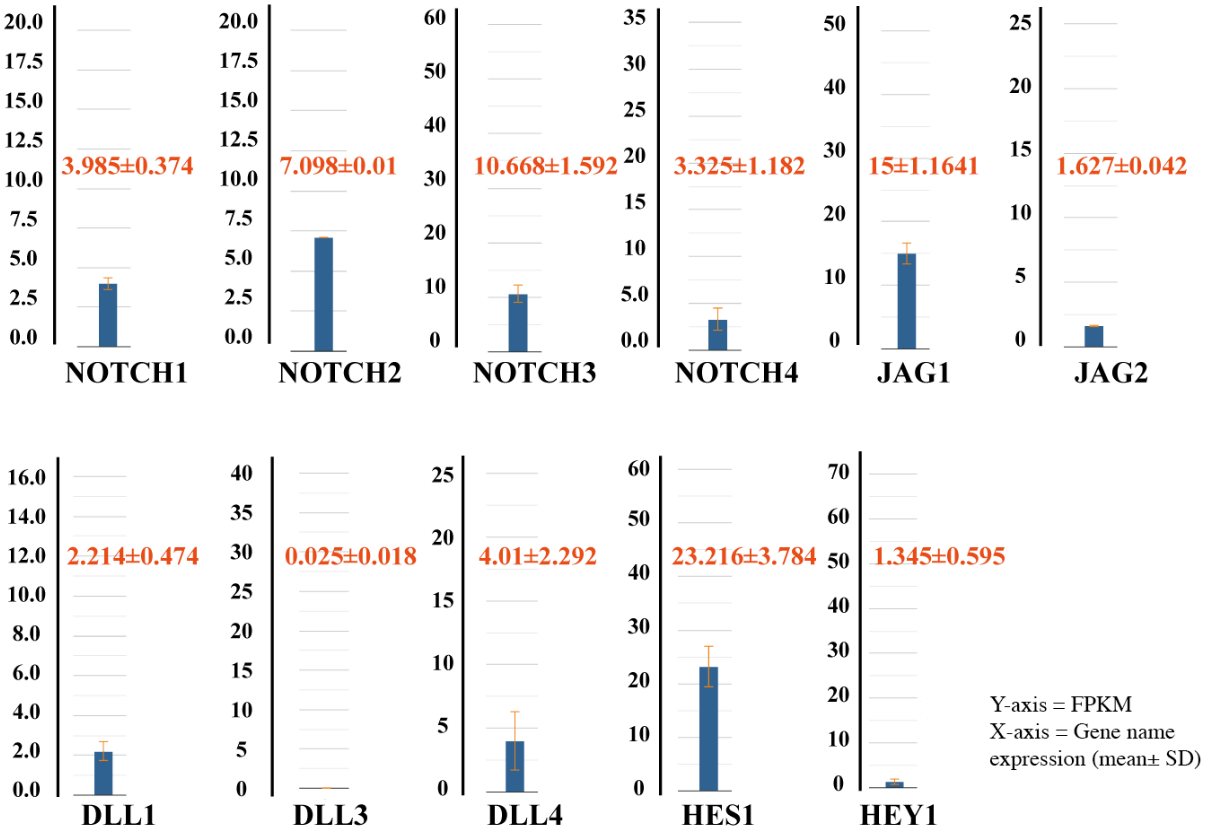

Figure S10. The expression level of target genes in normal bladder tissuse. RNA-sequencing (RNA-seq) by the National Center for Biotechnology Information (NCBI, USA) (https://www.ncbi.nlm.nih.gov/pmc/, accessed on Dec.13. 2020), (BioProject: PRJEB4337, *n* = 95, human) (Figure S6) [81,82]. The mean ± SD of the expression level were listed in the figures.

Table S6. The OS/DFS analysis of target genes and combinations in BCa.

Name	Cut-Off/OS	*p*-value	Name	Cut-Off/DFS	*p*-value	Name	Cut-Off/ Combination	*p*-value
N1	11.9	0.21	N1	11.6	0.32	N3+JAG1	23.7 (↓)/OS	0.047
N2	11.8 (↑↓)	0.017	N2	11.8 (↑↓)	0.013	N3+DLL4	15.7 (↓)/OS	0.036
N3	13.6 (↓)	0.0015	N3	13.5 (↓)	0.025	N3+JAG1+HES1	15.6 (↓)/OS	0.0024
N4	8.5	0.2	N4	9.5	0.45	N3+DLL4+HES1	8.5 (↓)/OS	0.0073
JAG1	14.8 (↓)	<0.0001	JAG1	14.3 (↓)	0.038	N3+JAG2	10.4 (↓)/DFS	0.018
JAG2	9.7	0.22	JAG2	9.7 (↓)	0.022	N3+JAG2+HES1	4.1 (↓)/DFS	0.015
DLL1	10.5	0.15	DLL1	8.3	0.49			
DLL3	5.2	0.48	DLL3	3.8	0.3			
DLL4	9.4 (↓)	0.0084	DLL4	8 (↑)	0.027			
HES1	11.6 (↑)	0.00094	HES1	11.8 (↑)	0.0023			
HEY1	12 (↓)	0.045	HEY1	11.5 (↓)	0.046			

NOTCH1 (N1), NOTCH2 (N2), NOTCH3 (N3), NOTCH4 (N4); (↑) means the case with high expression was associated with prolonged OS; (↓) means case with low expression was associated with prolonged OS. (↑↓) means the risk or advantageous factor was separated into different time periods.

Table S16. The overall survival analysis of target genes in BCa based on HPA.

Name	cut-off value	*p*-value	Cases in high expression group	Cases in low expression group
NOTCH1	11.7	0.088	84	322
NOTCH2	4.46 (↑↓)	0.0073	228	178
NOTCH3	27.73 (↓)	0.002	177	229
NOTCH4	2.33	0.31	97	309
JAG1	29.27 (↓)	0.0014	82	324
JAG2	2.79	0.22	310	96
DLL1	1.97	0.17	211	195
DLL3	0.28	0.3	117	289
DLL4	3.04 (↓)	0.0055	118	288
HES1	41.65 (↑)	0.00002	298	108
HEY1	5.37	0.1	89	317

"↑" means the case with high expression associate with prolong prognosis; "↓" means case with high expression associate with poor prognosis. "↑↓" means the risk or advantageous factor was separated into different period.
